# Machine learning–directed massively parallel programmable nucleic acid amplification

**DOI:** 10.1126/sciadv.aec9175

**Published:** 2026-03-25

**Authors:** Zhi Weng, Wenle Huang, Yi Wu, Xuehao Xiu, Hui Lv, Fei Wang, Xiaolei Zuo, Chunhai Fan, Ping Song

**Affiliations:** ^1^ International Peace Maternity and Child Health Hospital, School of Medicine, Shanghai Jiao Tong University, National Center for Translational Medicine, Shanghai 200030, China.; ^2^ School of Biomedical Engineering, Zhangjiang Institute for Advanced Study and National Center for Translational Medicine, Shanghai Jiao Tong University, Shanghai 200240, China.; ^3^ Institute of Materiobiology, College of Sciences, Shanghai University, Shanghai 200444, China.; ^4^ State Key Laboratory of Synergistic Chem-Bio Synthesis, School of Chemistry and Chemical Engineering, New Cornerstone Science Laboratory, Frontiers Science Center for Transformative Molecules, Zhangjiang Institute for Advanced Study and National Center for Translational Medicine, Shanghai Jiao Tong University, Shanghai 200240, China.; ^5^ Institute of Molecular Medicine, Shanghai Key Laboratory for Nucleic Acids Chemistry and Nanomedicine, Renji Hospital, School of Medicine, Shanghai Jiao Tong University, Shanghai 200127, China.

## Abstract

Dynamic regulation of amplification efficiency is pivotal yet challenging in molecular diagnostics and DNA data storage. Here, we develop a thermodynamics-based approach to achieve continuous and precise modulation of nucleic acid amplification efficiency. By decoupling sequence specificity from hybridization energy regulation via a primer-tag compensation strategy, we demonstrate programmed amplification with high resolution (33 versus 81%). Leveraging 2483 experimental data, we constructed a machine learning model that improved prediction accuracy from *R*^2^ = 0.62 to = 0.86. In DNA data storage, this amplification strategy increases the density for information preview by nearly one order of magnitude and robust file steganography via differential amplification. In clinical validation, our method outperformed uniform amplification in cervical cancer RNA variant analysis, detecting rare RNA fusions and improving detection sensitivity by 100-fold under 10^4^ simulated sequencing depth. This programmable technique is anticipated to extend to single-cell sequencing and spatial transcriptomics, offering a powerful tool for molecular diagnostics and synthetic biology.

## Teaser

Machine learning–guided tunable DNA amplification enables intelligent DNA data storage and ultrasensitive molecular diagnostics.

## INTRODUCTION

Nucleic acid amplification is indispensable to molecular diagnostics (*1*–*3*), genome sequencing (*4*–*7*), and emerging DNA-based data storage (*8*–*11*). However, its further advancement is bottlenecked by the limited flexibly and accurately with which amplification efficiency can be tuned across heterogeneous templates (*12*, *13*). Current high-throughput protocols still operate in a “one-size-fits-all” mode: applying identical primers, uniform thermal conditions, and fixed enzyme concentrations to every target, inevitably sacrificing sensitivity for specificity or vice versa (*14*–*16*). Conventional strategies that modulate efficiency by globally shortening or lengthening the same primer set result in switch-like responses: A single-base elongation or truncation can shift yields from undetectable to full saturation (*16*). This binary behavior precludes programmable, template-specific tuning and cascades into poor detection limits, uneven coverage, and elevated error rates, particularly in massively parallel reactions.

The persistent binary nature of amplification becomes clearer when contrasted with nucleic acid hybridization. The same thermodynamic principles that transformed hybridization from an all-or-none process into a continuously tunable one could, in principle, be applied to polymerase chain reaction (PCR) (*17*–*19*). However, applying these principles to PCR remains challenging. First, amplification yield is governed by an extremely narrow parameter window: A single-base change in primer-template complementarity can shift yields from 0 to more than 90%, resulting in a sharp, nearly step-wise response curve. Second, the reaction couples primer-template hybridization with enzyme kinetics, deoxynucleoside triphosphate (dNTP) availability, and product inhibition, creating a high-dimensional parameter space that resists straightforward predictive models. Collectively, these factors have hindered the development of general framework for programmable and template-specific amplification.

To the best of our knowledge, no effective method for continuous and tunable control of amplification efficiency has been reported to date. Here, we introduce a machine learning–directed approach for massively parallel programmed nucleic acid amplification. We first developed a tag-primer architecture that incorporates an energy-compensating sequence to enable stepwise regression of amplification efficiency. This design mitigates the impact of initial sequence adjustments and substantially broadens the dynamic range of amplification, with hybridization reaction standard free energy change (Δ*G*°) expanded from 1.5 to 4.0 kcal mol^−1^. Using these tag primers as regulatory units, we constructed an ensemble learning model capable of accurately predicting and precisely controlling amplification yield. As a proof of concept, we applied our programmable amplification (PA) strategy to DNA data storage. By differentially modulating the amplification efficiency of primers during file random access, we achieved template-specific amplification, successfully implementing multilevel access control in DNA storage systems and advancing toward intelligent data retrieval. Furthermore, in clinical validation through cervical cancer RNA variant analysis, our method substantially outperformed uniform amplification, detecting rare RNA fusions while improving detection sensitivity by 100-fold.

## RESULTS

### PA overview and prediction model for amplification

**Fig. 1.**
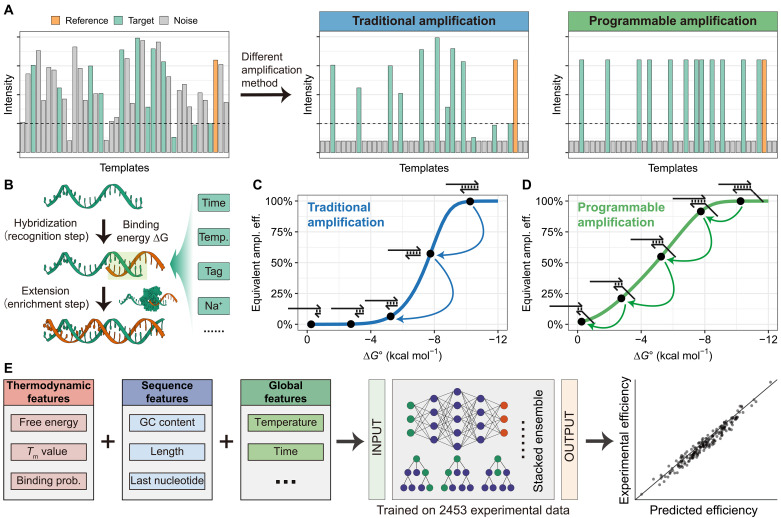
Overview of PA. (**A**) In conventional multiplex amplification systems, uniform amplification often leads to loss of low-frequency templates. In contrast, PA enables template-specific amplification tuning, facilitating both enrichment of low-abundance templates and on-demand multitarget analysis. (**B**) Workflow of PA experiment, comprising two key steps: signal recognition (nucleic acid hybridization) and enrichment step (enzymatic amplification). (**C**) Relationship between equivalent amplification efficiency (abbreviated Equivalent ampl. eff.) and primer hybridization thermodynamics in conventional methods. The narrow ∆*G* tuning range results in abrupt efficiency changes upon single-base modifications. (**D**) Thermodynamic regulation in PA shows an expanded ∆*G* tuning range, allowing precise programmability of amplification efficiency. (**E**) Schematic of the machine learning–assisted efficiency prediction model for PA.

On-demand multitemplate amplification is becoming increasingly important in complex sample analysis (*12*, *20*). Traditional amplification (TA) methods use uniform primers that produce consistent yields but offer limited flexibility in adjusting the relative proportions of different targets. This often leads to the underdetection of low-abundance species. In contrast, we developed PA to enable adjustable amplification efficiency for different targets, achieving on-demand amplification of multiple targets while maintaining precise analysis, particularly for low-abundance targets (Fig. 1A). The process primarily consists of two stages: molecular recognition involving hybridization between nucleic acid primers and templates, which determines reaction specificity (Fig. 1B). The hybridization standard free energy (Δ*G*) serves as an indicator of reaction efficiency and specificity (*16*, *18*, *21*), influenced by multiple factors including sequence information, temperature, and salt concentration—all of which contribute to subsequent model training. The second stage involves enzymatic amplification after primer-template binding, which determines the enrichment yield for subsequent hybridization and amplification. These two processes are progressively amplified over multiple PCR cycles, resulting in gradually diverging amplification efficiencies (Fig. 1, C and D). Of note, amplification efficiency denotes the fraction of template molecules duplicated per cycle with a theoretical maximum efficiency of 100%. The equivalent amplification efficiency represents the mean efficiency averaged across all cycles. Amplification yield is defined as the ratio of the actual product quantity to the theoretical maximum product quantity attainable after a given number of cycles.

Notably, conventional amplification systems show limited tunability when attempting to modulate efficiency through primer shortening, with single-base modifications often causing abrupt efficiency drops (Fig. 1C). To overcome this limitation, our PA system incorporates a tag-mediated regulation mechanism that sustains high equivalent amplification efficiency even under suboptimal Δ*G* conditions, substantially broadening the available dynamic range (Fig. 1D). Furthermore, to accurately predict equivalent amplification efficiency and meet the requirements for on-demand multitemplate amplification, we further developed a machine learning model incorporating multiple parameters of primer hybridization and amplification, such as ∆*G*, reaction time, temperature, and GC content, enabling accurate prediction of primer amplification yield (Fig. 1E). This comprehensive approach not only solves the problem of low-abundance target detection but also establishes a framework for precision-controlled nucleic acid amplification.

### Theoretical modeling and validation of PA

**Fig. 2.**
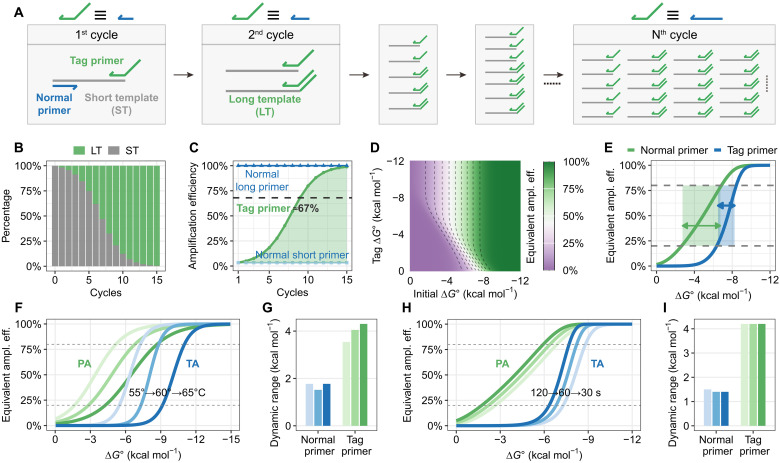
Theoretical modeling of PA. (**A**) Schematic of PA using tag primer. (**B**) Ratio variation between LT and ST and (**C**) corresponding equivalent amplification efficiency across PCR cycles, demonstrating results for primers with initial Δ*G*° = −6 kcal mol^−1^ and tag Δ*G*° = −12 kcal mol^−1^. As cycle number increases, LT proportion progressively rises. Because of stronger binding between LT and primers, the per-cycle equivalent amplification efficiency gradually “rebounds” to normal levels. As a result, the tag-primer system is equivalent to a conventional system (both FP and RP) with 67% efficiency (black dashed line). This “efficiency rebound” mechanism mitigates the impact of initial Δ*G* adjustments, thereby expanding the primer’s dynamic regulation range. (**D**) Equivalent amplification efficiency of primers with varying initial Δ*G*° and tag Δ*G*° values. More negative tag Δ*G*° values correspond to greater dynamic range expansion. (**E**) Comparison between tag primers (tag Δ*G*° = −12 kcal mol^−1^) and conventional primers across different initial Δ*G*° values, showing substantially broader dynamic range for tag primers (defined as the *x*-axis range corresponding to 20 to 80% efficiency values: blue, 1.75; green, 4.0). (**F**) Free energy–efficiency response curves and (**G**) dynamic range comparison for tag primers versus conventional primers at different temperatures. Data were fitted from 1000 sequences accounting for sequence-specific Δ*G*° temperature dependencies. Tag primers consistently exhibit larger dynamic ranges across temperatures. (**H**) Free energy–efficiency response curves and (**I**) dynamic range comparison at different extension times. Tag primers maintain superior dynamic ranges regardless of extension time.

To address the limited dynamic range and poor tunability of conventional primers, we developed a tag-primer design for PA. The key innovation involves incorporating a noncomplementary sequence at the 5′ terminus that provides progressive energetic compensation over amplification cycles, thereby enabling precise control of total amplification yield. This system gives rise to two template types: the initial short template (ST) and the extended long template (LT). Because of differential binding affinities, tag primers exhibit distinct amplification efficiencies for these templates. As cycles progress, LT proportion gradually increases, causing the overall amplification efficiency to asymptotically approach 100% (Fig. 2A). This efficiency restoration phenomenon mitigates the impact of single-base variations, substantially expanding the reaction’s dynamic range for fine-tuned amplification control.

We first established a theoretical model integrating primer hybridization and polymerase extension to validate the feasibility of tag primer (Supplementary Text and figs. S1 to S3). For a primer with an initial standard binding free energy Δ*G*° = −6 kcal mol^−1^ (calculated at 60°C, 0.18 M Na^+^) and a tag region with initial standard binding energy of Δ*G*° = −12 kcal mol^−1^, the proportion of LT increased from 0% at cycle 0 to 25% by cycle 7, exceeding 90% after cycle 13 (Fig. 2B). Correspondingly, the amplification efficiency rose from near 0 to 100%, matching the cumulative output of a conventional primer with 67% efficiency over 15 cycles (Fig. 2C and fig. S4). Systematic simulations revealed that rational design could achieve any desired efficiency within this continuum (Fig. 2D). Notably, increasing the binding strength of the tag Δ*G* substantially expanded the dynamic range compared to conventional primers (tag Δ*G*° = 0 kcal mol^−1^; Fig. 2, D and E).

Additional parameters like annealing temperature and extension time also modulate amplification efficiency (fig. S5). We therefore systematically evaluated their effects on dynamic range. Temperature primarily affects sequence-specific Δ*G* (fig. S6). Using Δ*G*° referenced to 60°C, we simulated efficiency variations across temperatures. While temperature dramatically shifted equivalent amplification efficiency curves (e.g., 57.8% at 60°C versus 95.6% at 55°C versus 15.2% at 65°C), it minimally affected conventional primers’ dynamic range. Tag primers showed slight range expansion in dynamic range at elevated temperatures (Fig. 2F) and consistently outperformed conventional primers across all conditions (Fig. 2G and fig. S7). Extension time exerted a weaker influence on both efficiency distribution (Fig. 2H) and dynamic range (Fig. 2I), although tag primers maintained superior tunability across all conditions.

### Experimental validation and performance characterization of engineered PA

**Fig. 3.**
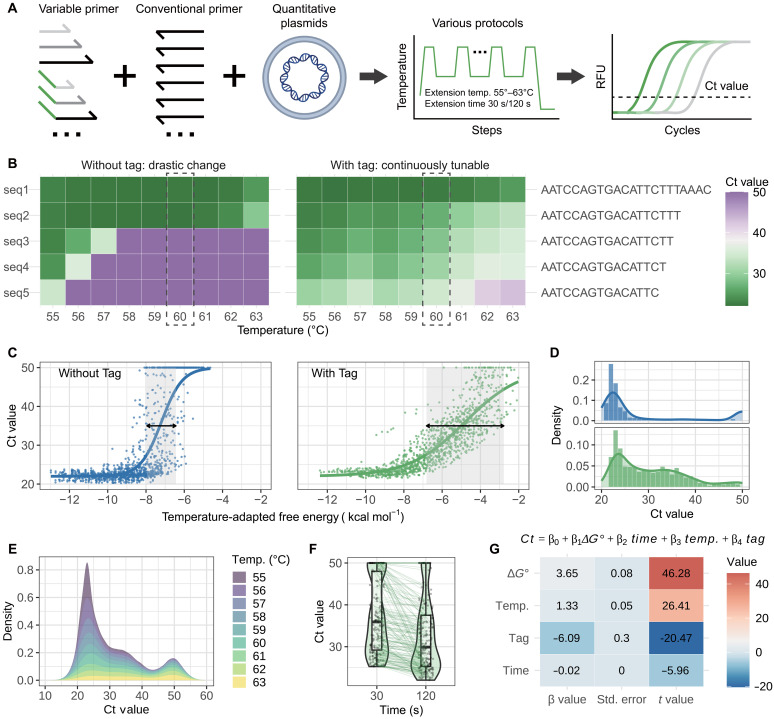
Experimental validation and performance analysis of tag-primer designs. (**A**) Amplification data collection using quantified plasmids (6000 copies/μl) with various primer designs and reaction protocols. Reactions showing no amplification within 50 cycles were recorded as Ct = 50. (**B**) Comparative efficiency modulation between tag primers and conventional primers through sequence truncation. Representative primer sets (including both types) were progressively shortened and tested at different temperatures (Ct values shown). The right panel displays the shared template-binding region. Conventional primers exhibit abrupt Ct transitions (green to purple), while tag primers demonstrate gradual, tunable efficiency shifts through combined sequence truncation and temperature adjustment. (**C**) Efficiency distributions versus free energy (calculated at 55° to 63°C). Tag primers show substantially broader dynamic ranges. (**D**) Ct distributions of conventional versus tag-primer reactions. Conventional primers yield bimodal extremes (low or high efficiency), whereas tag primers produce a near-uniform continuum across intermediate Ct values, indicating programmable, gradient-level amplification control. (**E**) Temperature-dependent Ct distributions. Higher temperatures induce right-skewed distributions due to reduced amplification efficiency. (**F**) Extension time effects. Longer extensions marginally improve efficiency (lower Ct values). For clarity, only data with Ct values greater than 25 at 30 s are shown for comparison. (**G**) Parameter influence ranking via linear regression. Tag presence dominates efficiency modulation, followed by free energy and temperature.

Following theoretical validation of primer-tag feasibility, we compiled experimental data from 2483 independent reaction records (TA: *n* = 1255; PA: *n* = 1228) to compare both primer systems. Data were generated by targeting multiple plasmid sites using various primer with varied configurations (with/without tags, sequence truncations) under different reaction protocols. To streamline subsequent analysis and prediction, we typically modified either the forward (FP) or reverse primer (RP) while maintaining the other as a conventional primer (Δ*G*° ≈ −12 kcal mol^−1^, efficiency ≈ 100%), using cycle threshold (Ct) values as a proxy for amplification yield (Fig. 3A).

Conventional primers exhibited drastic efficiency reduction upon single-base truncations (theoretical median efficiency drop: 81%), with experimental Ct values showing discontinuous jumps [e.g., 22 (green) to 50 (purple) at 60°C]. In contrast, tag primers enabled gradual efficiency modulation—Each truncation step induced modest Ct increments (median ΔCt ≈ 2 cycles), with a theoretical median efficiency drop of 33% (Fig. 3B and figs. S8 and S9). Curve fitting of Ct values against Δ*G* revealed that tag primers achieved a 4.0 kcal mol^−1^ dynamic range with 2.7-fold wider than conventional primers (1.5 kcal mol^−1^; Fig. 3C and figs. S10 and S11). This expanded tunability was further reflected in Ct value distributions: Conventional primers clustered at extreme values (Ct = 22 or 50), whereas tag primers showed a uniform distribution across intermediate Ct values (Fig. 3D), further confirming precise programmable control. To confirm the generalizability of tag strategy, we evaluated four distinct tag sequences with different GC content across multiple temperatures and primer lengths (fig. S12). All four tag sequences demonstrated consistent continuous tunability, maintaining gradual Ct modulation across different temperatures despite their sequence diversity. This robustness confirms that the PA strategy is sequence-independent and relies primarily on thermodynamic principles rather than specific sequence features, establishing broad applicability for primer design across diverse sequence contexts.

Beyond programmable tunability, tag primers demonstrated superior specificity in discriminating single-nucleotide variations. Systematic evaluation across three amplicons with primers containing single-base mismatches at varying positions revealed that tag primers exhibited substantially larger ΔCt differences between matched and mismatched scenarios compared to conventional primers (mean ΔCt: 12.0 versus 3.2 cycles; fig. S13). This enhanced discrimination arises from the truncated binding region in tag primers, which amplifies the energetic penalty of single-base mismatches that compounds exponentially over iterative PCR cycles, enabling robust discrimination between highly similar templates in multiplexed applications.

Having established tag primers’ tunability and specificity, we further characterized how reaction conditions affect amplification performance beyond sequence design. Temperature-dependent analysis across the full dataset indicated that higher temperatures reduced amplification efficiency, manifesting as right-skewed Ct distributions with elevated means, primarily attributable to the Δ*G* = Δ*H* – *T*Δ*S* relationship (Fig. 3E and figs. S14 and S15). In contrast, longer extension times marginally reduced Ct values (Fig. 3F and fig. S16).

To quantitatively evaluate the relative contributions of different parameters to Ct values, we performed linear regression analysis to determine weighting coefficients. Tag incorporation showed the strongest effect, reducing Ct by 6.1 cycles compared to conventional primers. Thermodynamic stability (Δ*G*) exerted the next largest influence, increasing Ct by 3.7 ± 0.1 per kcal mol^−1^. Temperature demonstrated moderate impact (1.3 ± 0.1 Ct elevation per °C), while extension time had the weakest effect (0.02 ± 0.01 Ct decrease per second; equivalent to 1.8 cycles from 30 to 120 s). Separate regression analyses revealed attenuated parametric influences in tag primers compared to conventional designs, further confirming tag primers’ enhanced tunability (Fig. 3G and figs. S17 and S18).

### Precise prediction of amplification efficiency using ensemble learning models

**Fig. 4.**
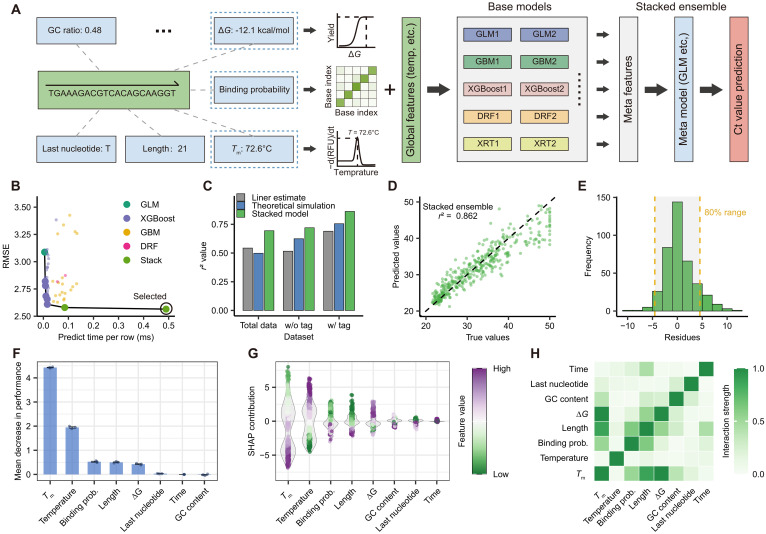
Machine learning models for predicting amplification efficiency. (**A**) Architecture of the ensemble learning–based prediction model. Sequence-specific biophysical features and global reaction parameters (temperature and time) were input into multiple base models, with meta-model stacking generating the final Ct value predictions. (**B**) Pareto plot comparing root mean square error (RMSE) and prediction time across models. The selected stacked model (black circle) achieved the lowest RMSE. (**C**) Performance comparison of different models across datasets. The “With tag” dataset showed superior prediction accuracy for all models, with the stacked model performing best within each dataset. (**D**) Scatter plot of predicted versus experimental values for the stacked model (*r*^2^ = 0.862). (**E**) Residual distribution with yellow dashed lines indicating the 80% confidence interval. Most predictions fell within ±4.5-cycle error range. (**F**) Feature importance ranking and (**G**) SHAP analysis. Thermodynamic parameters (*T*_m_, temperature, and binding probability) dominated predictive power. (**H**) Feature correlation analysis showing strong *T*_m_-Δ*G* interdependence, explaining Δ*G*’s lower independent ranking due to information redundancy.

Accurate prediction of amplification efficiency is essential to guide primer design and minimize experimental optimization (*19*, *22*, *23*). However, conventional theoretical models that primarily rely on global thermodynamic parameters such as Δ*G*°, temperature, and extension time often exhibit poor predictive performance. In our preliminary analyses, primers with identical Δ*G*° under the same reaction conditions exhibited substantial variation in amplification efficiency, highlighting the importance of sequence-specific features beyond simplified thermodynamic frameworks (Fig. 3B). To address this, we developed a machine learning model integrating diverse predictive factors, which include thermodynamic parameters, primer sequence biophysical characteristics, and global reaction conditions. By combining these heterogeneous features with tag-primer designs, our approach substantially improved the prediction precision (Fig. 4A and figs. S19 and S20).

In our framework, we used an ensemble learning architecture that leverages multiple base learners, including generalized linear models (GLM), gradient boosting machines (GBM), random forest (RF), and XGBoost. In the first tier, multiple submodels were trained for each algorithm type using 70% of the dataset (TA: *n* = 1255; PA: *n* = 1228). Optimal performers or randomly selected subsets were then used to generate meta-features for the second-tier stacked model (Fig. 4A). This ensemble approach effectively compensated for the limitations of individual models, achieving superior accuracy with the lowest root mean square error among all tested methods (Fig. 4B) while maintaining practical inference speeds (~0.5 ms per sequence). Most notably, when combined with tag-primer designs, the model achieved a prediction precision of *R*^2^ = 0.862 compared to 0.624 for conventional theoretical models (Fig. 4, C and D, and figs. S21 to S23). Residual analysis further confirmed that 80% of predictions fell within ±4.5 cycles of experimental values (Fig. 4E and fig. S24), establishing a robust foundation for precision amplification control.

Feature importance analysis identified key thermodynamic parameters as the dominant predictors across both base learners and the stacked model. These included melting temperature (*T*_m_), reaction temperature, binding probability, and Δ*G*°, all consistently contributing the strongest predictive power (Fig. 4F and figs. S25 and S26). This aligns with the kinetic saturation principle: Given sufficient reaction time (30 to 120 s), thermodynamic stability ultimately governs amplification efficiency. SHAP (SHapley Additive exPlanations) value analysis further validated thermodynamic dominance of thermodynamics in model decisions (Fig. 4G). The reduced independent contribution of ∆*G* may be due to information overlap with correlated features like *T*_m_. This interpretation was corroborated by strong *T*_m_-Δ*G*° correlation in SHAP-based feature dependence plots (Fig. 4H).

### Development of an intelligent DNA storage operating system

**Fig. 5.**
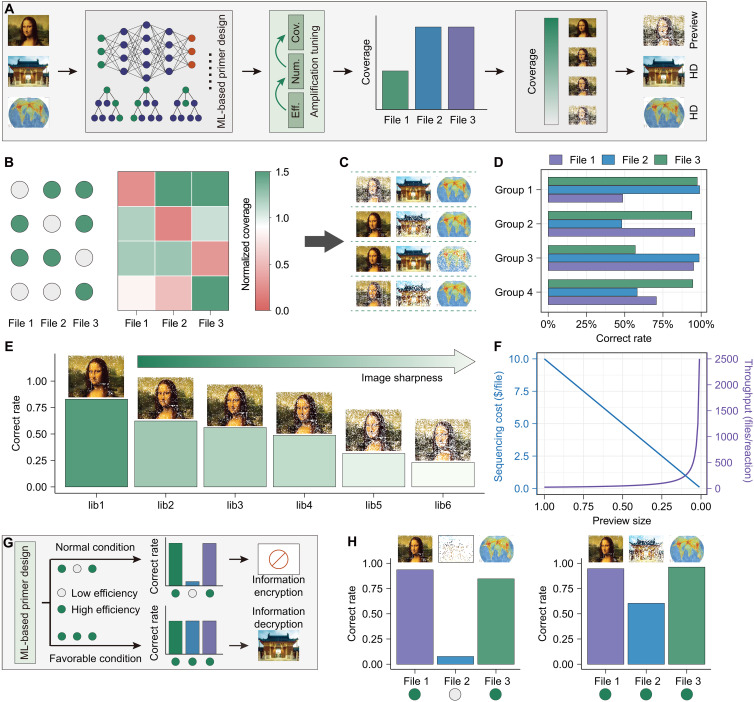
Machine learning (ML)–guided intelligent DNA storage system with personalized amplification control. (**A**) Schematic of the intelligent DNA storage operating system (map image credit: ICAO GIS). Under ML model guidance, the system modulates file-specific amplification efficiencies through optimized primer combinations and reaction condition adjustments, enabling differential molecular copy number control and customized readout outcomes within a single access operation. (**B** to **D**) File-specific (B) sequencing coverage, (C) decoding results, and (D) decoding accuracy under different primer combinations (green circles: high-efficiency primers; gray circles: low-efficiency primers). Results demonstrate programmable readout customization through primer efficiency regulation. (**E**) Continuous image clarity modulation via primer efficiency adjustment. (**F**) Cost-throughput optimization through preview-level control. Simulation assumes 25 M reads per run (1 M reads required for full file decoding), showing how PA increases throughput while reducing per-file costs at fixed sequencing capacity. (**G**) Condition-dependent file encryption/retrieval mechanism. Identical primers exhibit divergent amplification efficiencies under different reaction conditions, enabling cryptographic functionality. (**H**) Experimental validation showing file readout (left: 63°C, 30 s; right: 50°C, 120 s) with corresponding decoding accuracies.

DNA storage has emerged as a transformative solution for escalating data storage demands, leveraging DNA’s exceptional attributes including ultrahigh storage density, extended half-life, and minimal energy requirements (*24*–*26*). Nucleic acid amplification serves as a pivotal technology for file random access within this framework. The inherent complexity of DNA-encoded binary sequences presents unique challenges for amplification fidelity, making it an ideal test bed for our methodology. We have adapted our approach not only for conventional file retrieval but also engineered an intelligent access system capable of user-customized output (Fig. 5A).

The system operates through strategic primer design and reaction condition optimization guided by our predictive models. By differentially regulating amplification efficiencies across files, we generate distinct molecular copy number distributions that translate into variable sequencing coverage. This coverage modulation enables (i) simultaneous multifile access with customized readout quality and (ii) intelligent resource allocation based on user-defined priorities (Fig. 5, B to D). Experimental validation demonstrated that high-efficiency primers produced near-perfect decoding accuracy (≈100%), while low-efficiency primers generated “preview-mode” outputs (≈50% accuracy) at identical sequencing depths—effectively enabling tiered data access within a single run, with preview mode enhancing storage density by approximately an order of magnitude.

Further investigation of a fixed dual-file system revealed continuous clarity modulation (83 to 23% accuracy) through gradual primer efficiency reduction (Fig. 5E). This precision control enables dramatic resource optimization: preview-quality images required two orders-of-magnitude less sequencing coverage than full-resolution decoding under fixed total sequencing capacity (Fig. 5F). While extending this strategy to multiplex scenarios, increasing file numbers minimally affects PA’s tuning capability. However, the corresponding growth in primer pool size elevates cross-reactivity risks, particularly dimer formation, which may compromise modulation precision (fig. S27). Nevertheless, these challenges can be mitigated through stringent dimer screening and primer orthogonality validation during design.

Beyond primer-based control, we implemented condition-dependent file encryption through thermodynamic tuning. Designed “hidden” files exhibited near-zero amplification (7% accuracy) under standard conditions (63°C, 30s) but achieved normal detection (60% accuracy) when switched to optimized parameters (50°C, 120 s) (Fig. 5, G to H). Although decoded images showed partial quality reduction, the stark contrast between conditions conclusively established condition-triggered file access as a viable paradigm.

### PA for low-abundance RNA variant analysis

High-throughput sequencing has become a cornerstone technology in molecular diagnostics, with its exceptional sensitivity, throughput, and multiplexing capacity enabling widespread clinical applications in disease detection and precision medicine (*27*, *28*). Among these techniques, targeted sequencing demonstrates particular value for RNA fusion detection and mutation screening due to its superior sequencing depth, specificity, and cost-effectiveness (*29*, *30*).

**Fig. 6.**
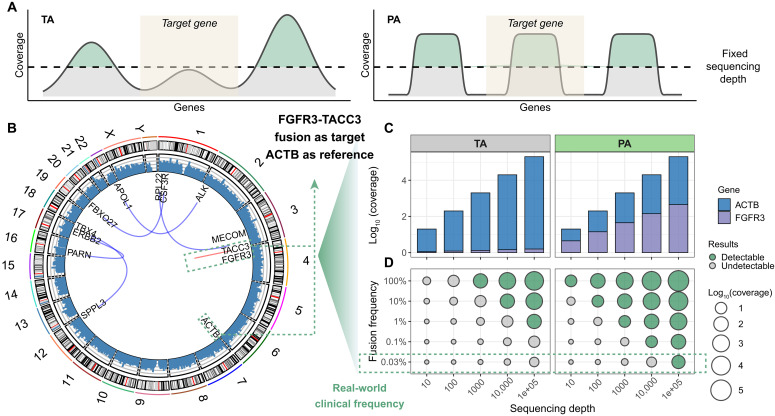
Application of PA in low-abundance RNA variant analysis. (**A**) Comparison of sequencing outcomes between different amplification methods. Conventional uniform amplification (left) results in overrepresentation of highly expressed reference genes, limiting detection of low-abundance targets due to read resource competition. In contrast, our tunable amplification strategy (right) enhances target gene representation through primer efficiency modulation, enabling effective detection at lower sequencing depths. (**B**) Circos plot of RNA sequencing data from clinical samples, demonstrating expression level variations spanning multiple orders of magnitude. (**C**) Read distribution patterns comparing conventional and tunable amplification methods across sequencing depths, using *FGFR3* (target) and *ACTB* (reference) for *FGFR3-TACC3* fusion detection. The tunable strategy substantially improves *FGFR3*’s relative abundance in sequencing data. (**D**) Detection sensitivity analysis across sequencing depths and fusion frequencies. The tunable approach achieves ≈100-fold lower detection limits at 10^4^ sequencing depths compared to conventional methods.

A critical challenge emerges from the substantial variation in RNA expression levels across genes, often spanning several orders of magnitude in clinical samples. Under uniform sequencing depth, this expression imbalance leads to inadequate coverage of low-abundance transcripts, increasing false-negative rates and compromising detection accuracy (Fig. 6A, left). Our tunable amplification strategy addresses this limitation by selectively modulating primer efficiencies to enhance representation of low-expression genes. This approach achieves balanced detection limits across targets without increasing total sequencing depth, substantially improving both sensitivity and diagnostic reliability (Fig. 6A, right).

We validated this system using clinical patient samples exhibiting natural expression variation (Fig. 6B). Focusing on FGFR3—a transmembrane tyrosine kinase receptor whose variants are implicated in cervical cancer, bladder cancer, and other malignancies (*31*, *32*)—we evaluated detection of *FGFR3-TACC3* fusion events using *ACTB* (a classical housekeeping gene) as reference. The 26-fold expression disparity between *FGFR3* and *ACTB* resulted in *FGFR3* capturing only 3.7% of sequencing reads under conventional amplification, with *ACTB* dominating the profile (Fig. 6C). At a simulated sequencing depth of 10,000×, the detection limit for TA was 10%, whereas our PA strategy achieved a detection limit of 0.1%, representing a 100-fold sensitivity improvement. Even at 100,000× sequencing depth, the minimum detectable fusion frequency of TA remained 1%, far exceeding the clinically required 0.03% threshold (Fig. 6D, left). Implementation of our PA strategy equalized gene representation, enabling reliable detection of 0.03% fusion events at equivalent sequencing depth and without additional costs (Fig. 6D, right).

## DISCUSSION

Our programmable nucleic acid amplification system enables precise control over amplification efficiency for different nucleic acid templates, representing a substantial advance over conventional uniform amplification methods. By combining this tunability with our machine learning prediction model, we have developed an amplification technology with broad application prospects across multiple fields.

In DNA data storage, our intelligent access system provides practical value by enabling differential readout of multiple files within a single access operation. Guided by machine learning predictions, the system regulates amplification efficiency to achieve customized file retrieval while optimizing resource utilization and enhancing information security. Notably, our approach generates variable-resolution outputs from a single high-definition file, eliminating the need to store separate preview versions. This design simplification improves system compatibility and scalability. Furthermore, continuous fine-tuning of amplification efficiency allows smooth gradation of readout clarity from full resolution to complete concealment, with flexible control through either primer design optimization or reaction condition adjustment.

The PA approach also demonstrates critical utility in clinical diagnostics, particularly for low-abundance RNA variants analysis. As evidenced in our *FGFR3-TACC3* fusion case study, this technology enables sensitive detection of rare RNA variants (down to 0.03% frequency) without increasing sequencing depth. This capability addresses a fundamental limitation in current RNA sequencing applications including bulk sequencing, single-cell analysis, and spatial transcriptomics, where expression level variations often span several orders of magnitude. By enabling on-demand efficiency adjustment for different targets, our method provides practical solutions for biomarker discovery and mechanistic studies while reducing false-negative rates in clinical testing.

Despite its promising performance, PA has certain limitations that warrant consideration. First, the machine learning model, trained on dataset from specific PCR systems, may exhibit limited generalizability across different experimental conditions or sample types. To ensure robust model evaluation, k-fold cross-validation can be used to verify stability and reliability across diverse data subsets. The model can serve as a pretrained foundation, enabling researchers to fine-tune it with limited data from their own experimental systems for rapid adaptation to different application scenarios. In addition, PA faces technical challenges common to conventional PCR. For instance, templates with extreme GC content, long amplicons, dimer formation, or extremely low starting copy numbers may compromise amplification efficiency and modulation precision. These limitations can be addressed through two complementary strategies: optimizing primer design to avoid extreme sequence features during the design phase, and improving reaction conditions through high-fidelity polymerases, high-GC buffers, or adjusted cycling parameters. Moving forward, collecting diverse external datasets and establishing model repositories tailored to different PCR systems will further validate and expand the applicability of PA approach.

While these limitations require continued attention, the fundamental advantages of PA—precise efficiency control and machine learning–guided optimization—position this technology for broad impact across molecular biology. Beyond these demonstrated applications, we anticipate that this PA technology will find broad utility in diverse molecular analysis scenarios including single-cell sequencing and spatial transcriptomics. The ability to prioritize specific targets through customized amplification efficiency control offers a more efficient and reliable solution for multiplex detection systems. Future work will focus on systematically addressing current limitations while further enhancing PA’s programmability and precision, ultimately broadening its applications in biomedical research and clinical diagnostics.

## MATERIALS AND METHODS

### Theoretical simulation of nucleic acid amplification

A kinetic model was developed to mechanistically simulate nucleic acid amplification, decomposing the PCR process into multiple repetitive cycles. Each cycle comprised primer-template hybridization and polymerase-mediated primer extension. The hybridization kinetics were parameterized using the law of mass action, with forward rate constants (*k*_f_) assumed to be ~10^6^ M^−1^ s^−1^ for oligonucleotides of several tens of bases. Reverse rate constants (*k*_r_) were estimated from the Δ*G*° via a thermodynamic relationship. The resulting system of ordinary differential equations was numerically solved using MATLAB’s stiff solver ode23s, with the output of one cycle serving as the initial state for the next. The model assumed constant enzyme activity, no secondary structures, and no side reactions. Detailed equations, parameter derivations, and simulation workflow are provided in Supplementary Text.

### Oligonucleotide synthesis and purification

Oligonucleotide pools were synthesized by Twist Bioscience (San Francisco, USA) and supplied as lyophilized DNA. All other individual DNA oligonucleotides were ordered from Sangon Biotech Co. (Shanghai, China). Unmodified oligonucleotides shorter than 59 nucleotides were purified using high-affinity purification, whereas those ≥59 nucleotides in length were purified by high-performance liquid chromatography.

### Nucleic acid amplification data acquisition and processing

Nucleic acid amplification experiments were conducted on a CFX96 Touch Real-Time PCR Detection System (Bio-Rad) using 96-well plates, with Blue SYBR Green Master Mix (YEASEN, catalog no. 11184ES03) as the reaction reagent. Plasmids containing multiple target sites were used as templates. For each target site, several primers were designed by base insertion/deletion and by including or excluding tag sequences. Primer pairs were assembled by combining one programmable primer (either FP or RP) with a conventional primer (Δ*G*° ≈ −12 kcal mol^−1^), thereby varying only a single primer within each experimental group to reduce complexity.

All reactions were performed at a fixed plasmid template concentration (6000 copies/μl). Each 10 μl reaction consisted of 5 μl of Blue 2× Master Mix, 1 μl of DNA template, 1 μl of FP (4 μM), 1 μl of RP (4 μM), and nuclease-free water to the final volume. A two-step cycling protocol was applied: initial denaturation at 95°C for 3 min, followed by 50 cycles of denaturation at 95°C for 10 s, and annealing/extension at a designated temperature for 30 s. Each primer set was tested under multiple annealing/extension temperatures, with one temperature applied per reaction plate.

For each condition, three parallel replicates were included. Upon completion of amplification, a standard melting curve analysis was performed. Melting curve profiles were inspected to exclude reactions with abnormal amplification peaks. To minimize the influence of outliers, the median Ct value from the three replicates was used as the final result for subsequent modeling.

### Model training and interpretability analysis

A regression model was developed to predict PCR amplification performance, formulated as the direct estimation of Ct values from primer sequence features and experimental conditions. The dataset comprised multiple primers tested under varying temperatures, extension times, and tag configurations. To prevent data leakage, data were split by primer identity (70% training, 30% validation), ensuring that all results for a given primer were assigned exclusively to one set. Three datasets were modeled separately: without tag, with tag, and the combined total dataset (including a binary “tag” feature).

Features included reaction parameters (temperature and time), primer properties (length, GC content, and last nucleotide), and thermodynamic stability (Δ*G*, *T*_m_, and average binding probability). Model training was performed using the H_2_O AutoML framework, generating multiple base learners (GLM, DRF, GBM, XGBoost, and XRT) and combining top models into a stacked ensemble for final prediction.

Interpretability analysis was conducted on the stacked ensemble to assess feature influence. Global importance was determined using model-agnostic permutation importance, local effects were quantified via SHAP values, marginal effects were visualized with partial dependence plots, and feature interaction patterns were evaluated using Pearson correlation of SHAP contributions. More details are provided in Supplementary Text.

### Random access of target files and NGS library preparation

To retrieve specific files, primer pairs were selected on the basis of the desired access pattern, allowing the use of programmable primers with tailored amplification efficiencies to achieve different retrieval outcomes. FPs and RPs for the chosen targets were mixed to a final concentration of 4 μM. Target sequences were amplified using Phusion DNA polymerase (Thermo Fisher Scientific, catalog no. F-530 L) in a 50-μl reaction containing 2 μl of the oligo pool, 2.5 μl of FP mix, 2.5 μl of RP mix, 0.5 μl of polymerase, 1 μl of 10 mM dNTPs, 10 μl of 5× high-fidelity (HF) buffer, and 31.5 μl of nuclease-free water. PCR was performed with an initial denaturation at 98°C for 2 min, followed by 12 cycles of 98°C for 20 s and 63°C for 10 s. The resulting amplicons were purified using a magnetic bead–based cleanup kit (Vazyme, catalog no. N411-02).

Adapter sequences were then appended in a second PCR step. For this, 15 μl of purified amplicon was combined with 2.5 μl of adapter FP mix, 2.5 μl of adapter RP mix, 0.5 μl of polymerase, 1 μl of 10 mM dNTPs, 10 μl of 5× HF buffer, and 18.5 μl of water, giving a total volume of 50 μl. Thermocycling was carried out with an initial 98°C for 2 min, followed by three cycles of 98°C for 20 s and 63°C for 10 s. PCR products were again bead-purified to remove residual primers and nucleotides.

The purified products were diluted 100-fold and used as templates in a quantitative PCR (qPCR) quantification assay. Each 10 μl of reaction contained 5 μl of Blue 2× Master Mix (YEASEN), 3 μl of diluted amplicon, 1 μl of N5 primer (5× diluted), and 1 μl of N7 primer (5× diluted). The qPCR program began with 95°C for 3 min, followed by 40 cycles of 95°C for 10 s and 60°C for 30 s. The obtained Ct values were used to determine the optimal cycle number for indexing.

For indexing, 15 μl of diluted amplicon was mixed with 1 μl of N5 index primer, 1 μl of N7 index primer, 0.5 μl of polymerase, 1 μl of 10 mM dNTPs, 10 μl of 5× HF buffer, and 21.5 μl of water, to a final volume of 50 μl. PCR was performed with an initial denaturation at 98°C for 2 min, followed by Ct + four cycles of 98°C for 20 s and 63°C for 10 s. Indexed products were purified, quantified using a commercial kit (Sangon, catalog no. N608301-0500), and pooled in equimolar amounts before next-generation sequencing.

### Data encoding and decoding

Digital image files were first divided into smaller information blocks, each assigned a unique address. These blocks were then converted into DNA sequences according to a predefined coding scheme, after which primer sequences were appended to both ends. The resulting oligonucleotide set was synthesized as a pooled library by a commercial DNA synthesis provider.

For data retrieval, sequencing results were first normalized to the same sequencing depth (typically 30× coverage) by down-sampling. The processed reads were then examined for correct primer sequences to confirm validity and identify the corresponding file. From each valid read, the address and payload regions were extracted, and payloads were grouped by their address labels. For each address, the most frequently occurring payload sequence—provided that it surpassed a predefined occurrence threshold—was retained for reconstruction. Any pixel positions where a valid payload could not be recovered were replaced with white pixels to preserve the structural layout of the decoded image.
