# Supplementary material for: Machine learning–directed massively parallel programmable nucleic acid amplification
Source: Sci Adv. 2026 Mar 25;12(13):eaec9175. doi: 10.1126/sciadv.aec9175 (PMC13015887; doi:10.1126/sciadv.aec9175)
Supplement: Supplementary file 1 — Supplementary Text Figs. S1 to S27 Tables S1 to S3 [file sciadv.aec9175_sm.pdf]

Science Advances
AAAS

Supplementary Materials for

Machine learning–directed massively parallel programmable nucleic acid amplification

Zhi Weng *et al.*

Corresponding author: Chunhai Fan, fanchunhai@sjtu.edu.cn; Ping Song, songpingsjtu@sjtu.edu.cn

Sci. Adv. **12**, eaec9175 (2026)
DOI: 10.1126/sciadv.aec9175

This PDF file includes:

Supplementary Text
Figs. S1 to S27
Tables S1 to S3

Supplementary Text

Supplementary Text 1. Theoretical simulation of nucleic acid amplification

To gain mechanistic insights into the amplification process, we constructed a kinetic model that decomposes nucleic acid amplification into multiple repetitive cycles, which collectively achieve exponential amplification. Each cycle consists of two primary reactions: nucleic acid hybridization between primer and template, and subsequent primer extension mediated by polymerase (Fig. S1).

The reactions considered in a single cycle are summarized below:

$$\mathrm{P1 + ST1} \underset{k_{off}1}{\overset{k_{on}1}{\rightleftharpoons}} \mathrm{hdDNA1}$$

$$\mathrm{hdDNA1} \overset{k_{on}5}{-} \mathrm{ST}$$

$$\mathrm{P2 + ST2} \underset{k_{off}2}{\overset{k_{on}2}{\rightleftharpoons}} \mathrm{hdDNA2}$$

$$\mathrm{hdDNA2} \overset{k_{on}5}{-} \mathrm{LT}$$

$$\mathrm{ST1 + ST2} \overset{k_{on}4}{-} \mathrm{ST}$$

$$\mathrm{P1 + LT1} \underset{k_{off}1}{\overset{k_{on}1}{\rightleftharpoons}} \mathrm{hdDNA3}$$

$$\mathrm{hdDNA3} \overset{k_{on}5}{-} \mathrm{LT}$$

$$\mathrm{P2 + LT2} \underset{k_{off}3}{\overset{k_{on}3}{\rightleftharpoons}} \mathrm{hdDNA4}$$

$$\mathrm{hdDNA4} \overset{k_{on}5}{-} \mathrm{LT}$$

$$\mathrm{LT1 + LT2} \overset{k_{on}4}{\longrightarrow} \mathrm{LT}$$

Applying the law of mass action, the system of ordinary differential equations governing species concentrations was established as follows:

$$\frac{d[ST1]}{dt} = -kf1*[ST1]*[ST2] - kf1*[ST1]*[LT2] - kf2*[ST1]*[P1] + kr2*[hdDNA1]$$

$$\frac{d[ST2]}{dt} = -kf1*[ST1]*[ST2] - kf1*[LT1]*[ST2] - kf3*[ST2]*[P2] + kr3*[hdDNA2]$$

$$\frac{d[LT1]}{dt} = -kf1*[LT1]*[ST2] - kf1*[LT1]*[LT2] -kf2*[LT1]*[P1] + kr2*[hdDNA3]$$

$$\frac{d[LT2]}{dt} = -kf1*[ST1]*[LT2] - kf1*[LT1]*[LT2] - kf4*[LT2]*[P2] + kr4*[hdDNA4]$$

$$\frac{d[ST]}{dt} = kf1*[ST1]*[ST2] + kf5*[hdDNA1]$$

$$\frac{d[LT]}{dt} = kf1*[LT1]*[ST2] + kf1*[ST1]*[LT2] + kf1*[LT1]*[LT2] +$$

$$kf5*([hdDNA2]+[hdDNA3]+[hdDNA4])$$

$$\frac{d[P1]}{dt} = -kf2*[ST1]*[P1] + kr2*[hdDNA1] - kf2*[LT1]*[P1] + kr2*[hdDNA3]$$

$$\frac{d[P2]}{dt} = -kf3*[ST2]*[P2] + kr3*[hdDNA2] - kf4*[LT2]*[P2] + kr4*[hdDNA4]$$

$$\frac{d[hdDNA1]}{dt} = kf2*[ST1]*[P1] - kr2*[hdDNA1] - kf5*[hdDNA1]$$

$$\frac{d[hdDNA2]}{dt} = kf3*[ST2]*[P2] - kr3*[hdDNA2] - kf5*[hdDNA2]$$

$$\frac{d[hdDNA3]}{dt} = kf2*[LT1]*[P1] - kr2*[hdDNA3] - kf5*[hdDNA3]$$

$$\frac{d[hdDNA4]}{dt} = kf4*[LT2]*[P2] - kr4*[hdDNA4] - kf5*[hdDNA4]$$

For oligonucleotide hybridization involving primers of several tens of bases, the forward rate constant (kf) is typically similar (~10^6 M^{-1} s^{-1}). Accordingly, the reverse rate constant (kr) can be inferred from the hybridization free energy (ΔG°) using the relationship:

$$\frac{kf}{kr} = e^{\Delta G^\circ/(RT)}$$

where R is the universal gas constant and T is the absolute temperature. This approach allows estimation of the kinetic parameters directly from thermodynamic stability, enabling simulation of reaction dynamics across multiple PCR cycles (**Fig. S**2).

To simplify the analysis, the following assumptions were applied: enzyme activity remains constant across cycles; secondary structures of primers and templates are neglected; and side reactions such as non-specific binding and mis-priming are ignored. The ODE system was numerically integrated using MATLAB's stiff solver "ode23s". Concentration profiles of all species were computed over a single cycle, and the final concentrations served as initial conditions for the subsequent cycle. By iterating across multiple cycles, the amplification curve was reconstructed.

Supplementary Text 2. Model Training and Interpretability Analysis

To systematically predict PCR amplification efficiency, we built a regression model that directly estimates the cycle threshold (Ct) value from primer features and experimental conditions. The target variable was the raw Ct value obtained from a BioRad real-time PCR system, using a plasmid template at a fixed concentration of 6000 copies/μL.

Dataset Construction and Splitting Strategy

We constructed a curated dataset consisting of multiple primer sequences tested across different temperatures, extension times, and primer configurations (with or without tag sequences). Each data point represents an average result of three parallel PCR experiments, with the corresponding Ct value recorded.

To ensure robust evaluation and avoid data leakage, we partitioned the dataset by primer sequence identity, such that all data related to a given primer (e.g., its performance under various temperatures or with/without a tag) were exclusively assigned to either the training or validation set, but never both. This strategy prevents the model from indirectly learning primer-specific characteristics during validation.

- **Training/Validation split ratio:** 70% / 30%.
- Three datasets were created for comparative modeling:
    - **Without tag:** containing only conventional primers;
    - **With tag:** containing primers extended with tag sequences;
    - **Total dataset:** combining both groups, with an additional binary feature ("tag") indicating tag presence.

Input Features

The selected features included both thermodynamic descriptors and sequence-level properties. For the with-tag and without-tag datasets, the model was trained using the following 8 features:

- **Temperature (°C):** Reaction temperature.
- **Time (s):** Extension time.
- **Length:** Primer length in nucleotides.
- **ΔG:** Gibbs free energy of primer hybridization (calculated at the standard reaction condition).
- **GC content (%):** Fraction of G and C nucleotides.
- **Tm (°C):** Predicted melting temperature.

- **Average binding probability:** Probability of primer-target binding based on equilibrium calculations.
- **Last nucleotide:** The 3' end base of the primer, encoded as a categorical feature.

For the total dataset, an additional categorical feature was included:

- **Tag:** Binary indicator of whether a tag was present (1) or not (0).

Model Training

All models were trained using the H2O AutoML framework. The prediction task was formulated as a regression problem. AutoML was configured to automatically train a variety of base models, including:

- Generalized Linear Models (GLM)
- Random Forest (DRF)
- Gradient Boosting Machines (GBM)
- Extreme Gradient Boosting (XGBoost)
- Extremely Randomized Trees (XRT)

Each base model was trained with default H2O settings, with early stopping and leaderboard-based ranking. The final ensemble model was a Stacked Ensemble, which combines the top-performing base learners through meta-learning. Model performance was evaluated using metrics such as R^2, Root Mean Square Error (RMSE), and Residual analysis.

Interpretability Analysis

To better understand the internal decision mechanisms of the Stacked Ensemble model and the contribution of individual features to amplification efficiency, we conducted a comprehensive interpretability analysis from multiple perspectives. This analysis combined global and local interpretability metrics using tools such as DALEXtra and custom visualization scripts. All analyses were performed on the final trained Stacked Ensemble model unless otherwise noted.

(a) Variable Importance Ranking

To identify globally influential features, we computed model-agnostic permutation feature importance values using the DALEXtra package. This method quantifies the increase in model error when each feature's values are randomly shuffled, thereby disrupting its relationship with the target (Ct). The resulting rankings highlighted Tm, Temperature, and average binding probability as consistently influential across datasets.

(b) SHAP (SHapley Additive Explanations) Analysis

To explore local interpretability, we calculated SHAP values using the DALEXtra::predict_parts() function with type = "shap" for a subset of validation samples. This was performed on the trained Stacked Ensemble model and visualized using summary beeswarm plots.

(c) Partial Dependence Plots (PDPs)

To evaluate the marginal effect of individual features on predicted Ct values, we utilized the built-in h2o.partialPlot() function from the H2O framework. This method computes the average model prediction while varying one feature and keeping others fixed, providing insight into feature influence on model output.

(d) Feature Correlation Analysis

To evaluate how features interact, we computed a feature interaction heatmap using pairwise Pearson correlation of SHAP contributions across all samples. This analysis revealed how pairs of features jointly influenced model output, highlighting possible collinearity or synergistic effects.

All analysis scripts used for model training, evaluation, and interpretability—including SHAP calculation, variable importance ranking, PDP generation, and feature correlation heatmap construction—are available in the supplementary github repository.

Supplementary Figure

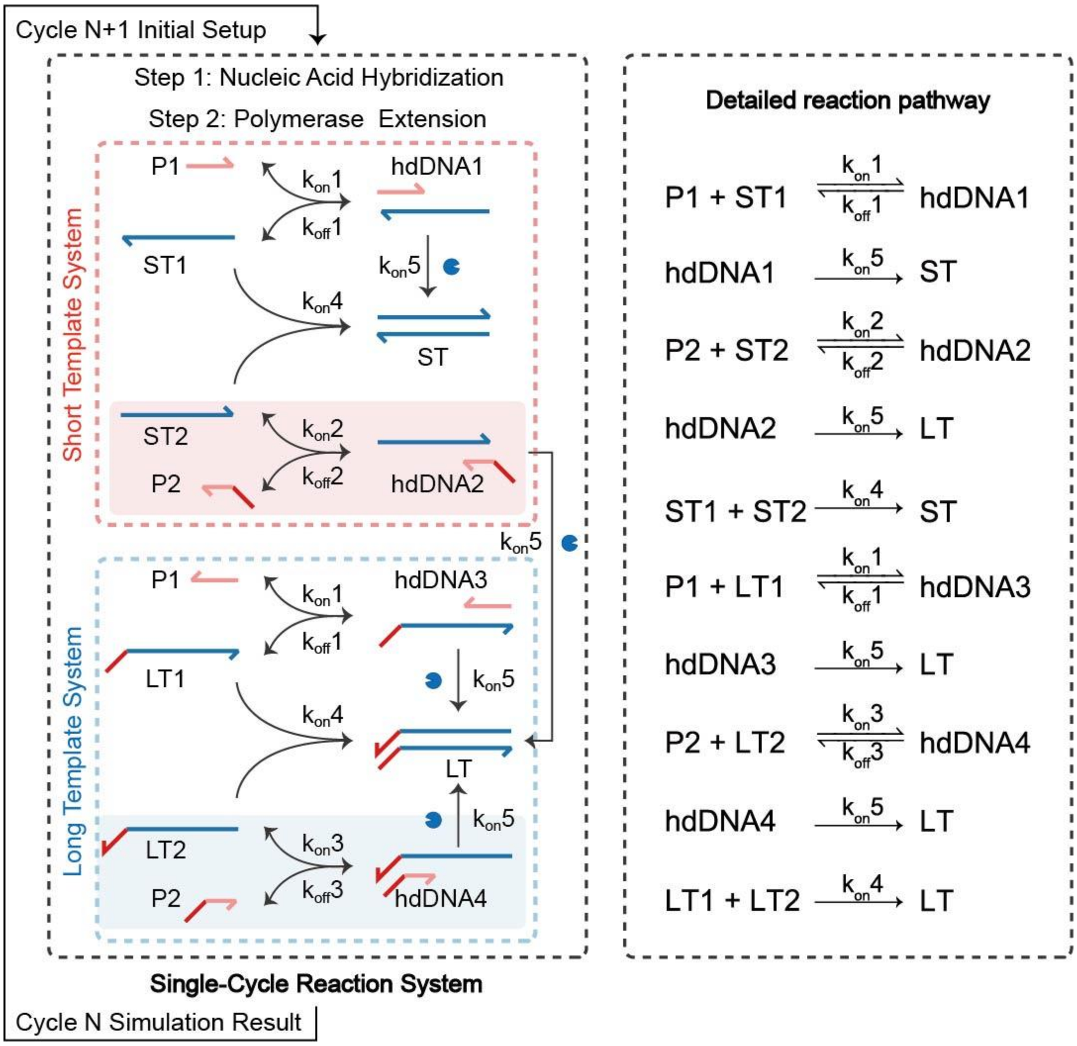

Fig. S1 | Schematic of the theoretical simulation.

The PCR process was modeled by decomposing multiple amplification cycles into sequential single reactions, where the output of one cycle served as the input for the next. Each reaction involved two major steps: nucleic acid hybridization and polymerase extension. Reaction pathways were translated into differential equations implemented in MATLAB, with parameters obtained from the literature, and solved using the ode23s stiff solver to generate the final simulation results.

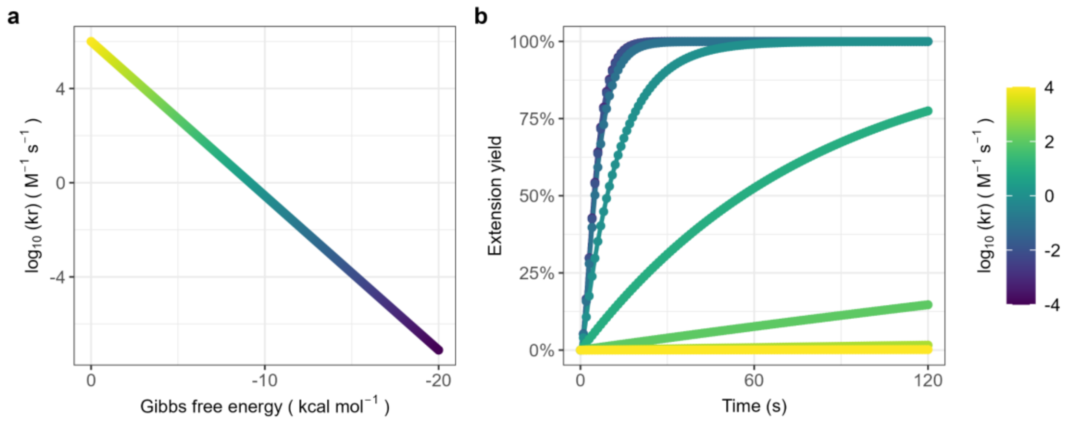

Fig. S2 | Theoretical simulation of reaction kinetics under varying free energies.

(a) Reverse rate constant (k_r) at different ΔG° values, assuming a fixed forward rate constant of kf = 10^6 M^{-1} s^{-1} and calculated as k_r = kf·e^(ΔG°/RT). (b) Simulated reaction kinetics at different k_r values. All simulations were performed at 60 °C.

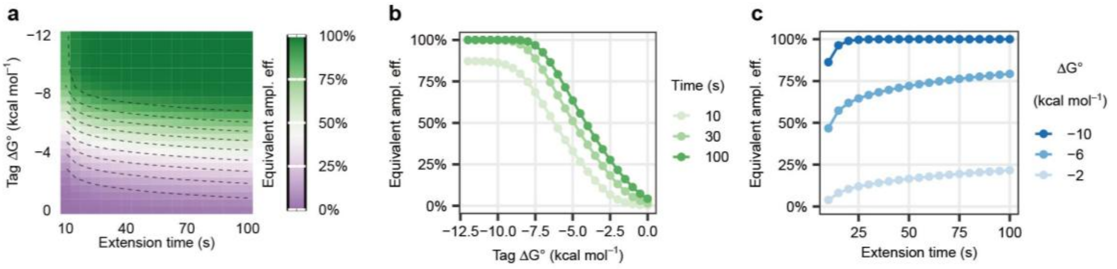

Fig. S3 | Influence of Gibbs free energy and extension time on Tag-primer efficiency.

(a) Heatmap showing primer efficiency across different Tag ΔG° values and extension times. (b) Efficiency variation curves at fixed extension times with varying Tag ΔG°. (c) Efficiency variation curves at fixed Tag ΔG° with varying extension times. At shorter extension times (e.g., less than the typical 30 s), kinetic factors exert significant influence on amplification efficiency. As extension time increases, the impact of kinetic factors gradually diminishes, while thermodynamic factors consistently play a dominant role throughout the amplification process.

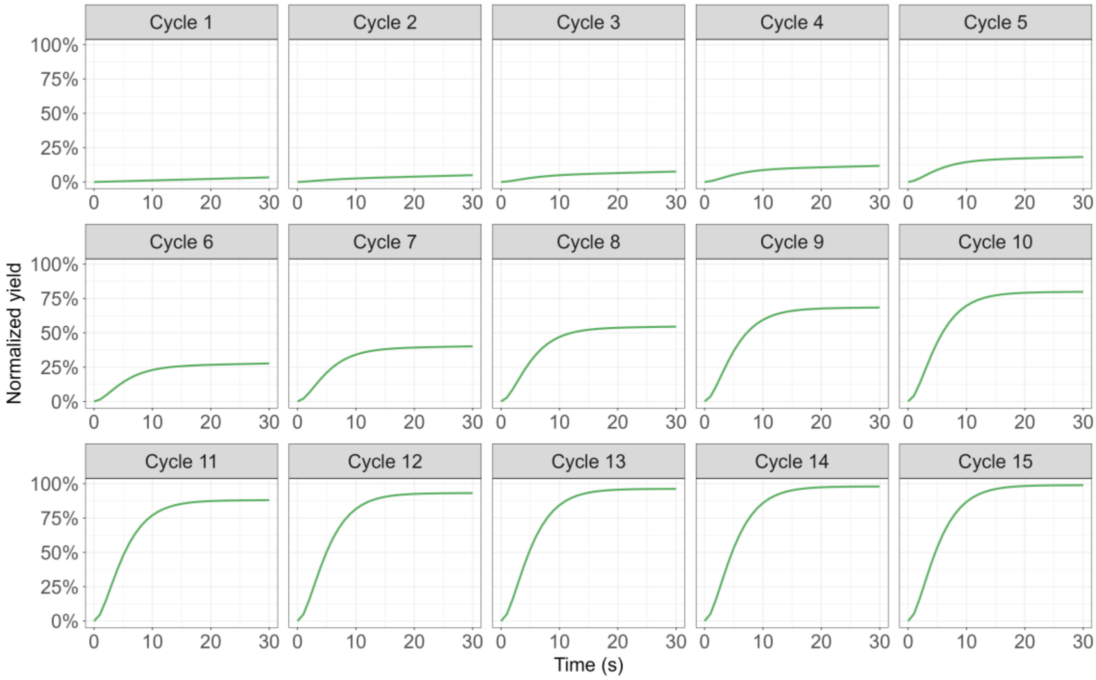

Fig. S4 | Primer-template kinetics within individual amplification cycles.

Simulation of a tag primer with an initial standard binding free energy $\Delta G° = -6$ kcal mol^{-1} and a tag energy of $\Delta G° = -12$ kcal mol^{-1}. Normalized yield is calculated based on the concentration of generated sequences at the end of an amplification cycle, normalized to the initial template concentration at the beginning of that cycle. The system achieves performance equivalent to a conventional primer with 67% efficiency over 15 cycles.

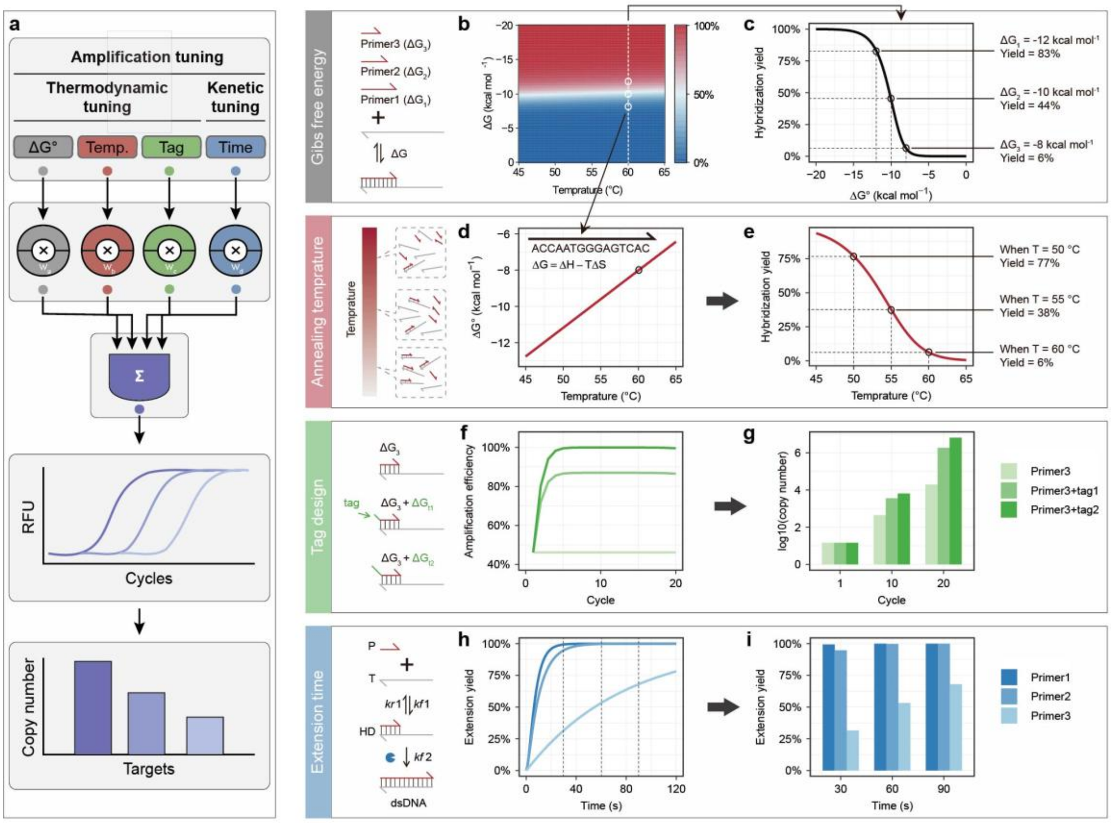

Fig. S5 | Theoretical validation of amplification tunability.

(a) Multiple factors act synergistically at the thermodynamic and kinetic levels to tune amplification efficiency, resulting in varying copy numbers. (b) Hybridization yield under different ΔG° values and temperatures. (c) Variation in hybridization yield at 60 °C as a function of ΔG°; hollow circles represent primers 1–3. (d) ΔG° of primer 3 at different temperatures and corresponding (e) hybridization yield. (f) Amplification efficiency of primer 3 with different tag sequences across amplification cycles and (g) the resulting copy numbers; ΔG° for tag1 and tag2 is −6 and −12 kcal mol^{-1}, respectively. (h) Amplification kinetics of different primers in a single cycle. (i) Final extension yields at different extension times. Unless otherwise specified, temperature was 60 °C.

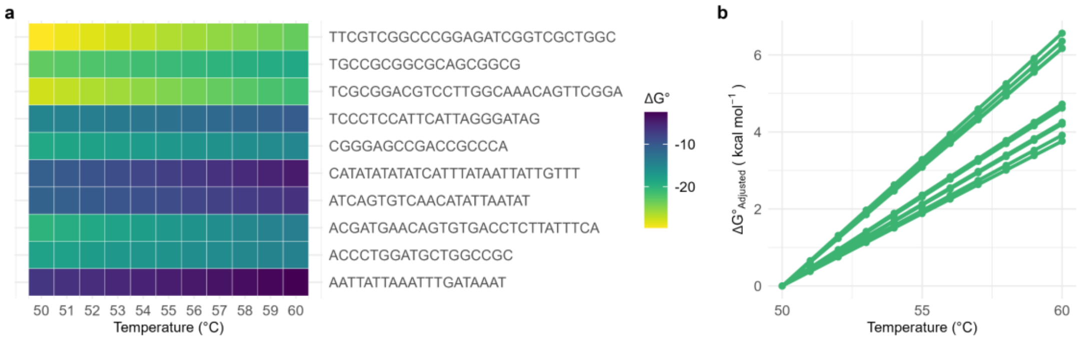

Fig. S6 | Effect of temperature on sequence hybridization free energy.

(a) Free energy of different sequences across varying temperatures. (b) Linear relationship between free energy and temperature for different sequences. $\Delta G_{adjusted}$ is calculated as the difference from the free energy at 50 °C for each sequence. Based on $\Delta G = \Delta H - T\Delta S$, the slope reflects entropy (ΔS), resulting in sequence-dependent trends.

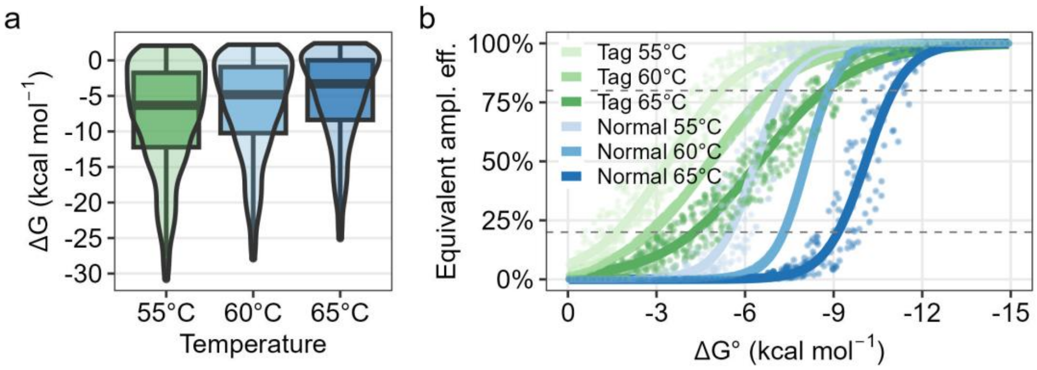

Fig. S7 | Temperature-dependent behavior of DNA sequences in amplification.

(a) Free energy distribution of different DNA sequences across three reaction temperatures (55°C, 60°C, and 65°C). (b) Simulated equivalent amplification efficiency profiles of sequences across temperature gradients. We generated 1,000 sequences with varying lengths and GC content, and calculated their free energies at different temperatures. Each data point represents a single sequence's equivalent amplification efficiency value at a specific temperature, with the x-axis showing the sequence's free energy under standard conditions (60°C). Fitted curves are based on scatter plot regression analysis.

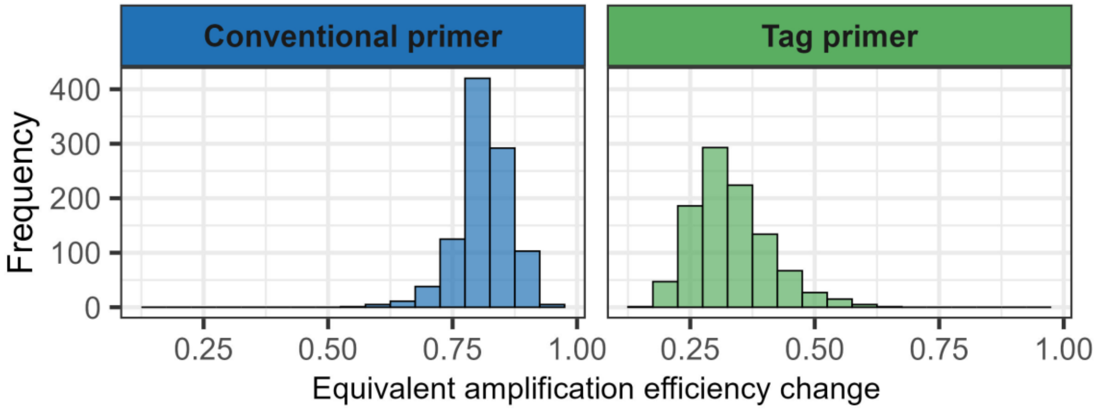

Fig. S8 | Efficiency response to single-base truncations in different primer designs.

Frequency histograms showing equivalent amplification efficiency changes after 3'-terminal single-nucleotide truncations for conventional primers and Tag-primers (for each, n = 1,000). Initial binding free energies were constrained within each design's dynamic regulation range. Efficiency differences (post-truncation vs. initial) were calculated from theoretical simulations. Median efficiency reduction: 81% (conventional primers) vs. 33% (Tag- primers).

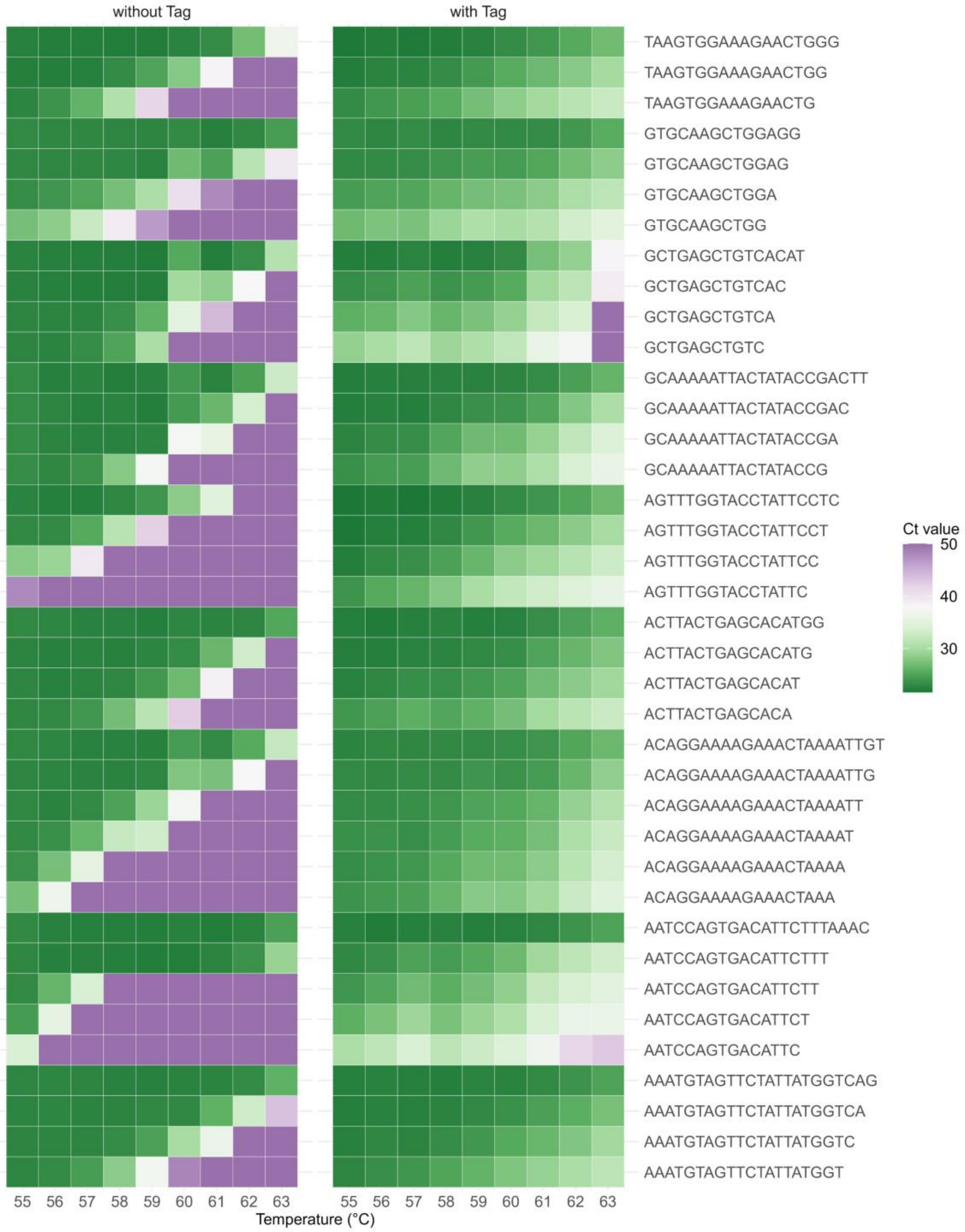

Fig. S9 | Comparison of amplification performance between tag primers and normal primers across various sequences and temperatures.

The right-side Y-axis labels display primer sequences representing the shared region between tag primers and normal primers. Compared with normal primers, tag primers exhibited more gradual and continuous Ct variations with sequence changes, reflecting improved tunability.

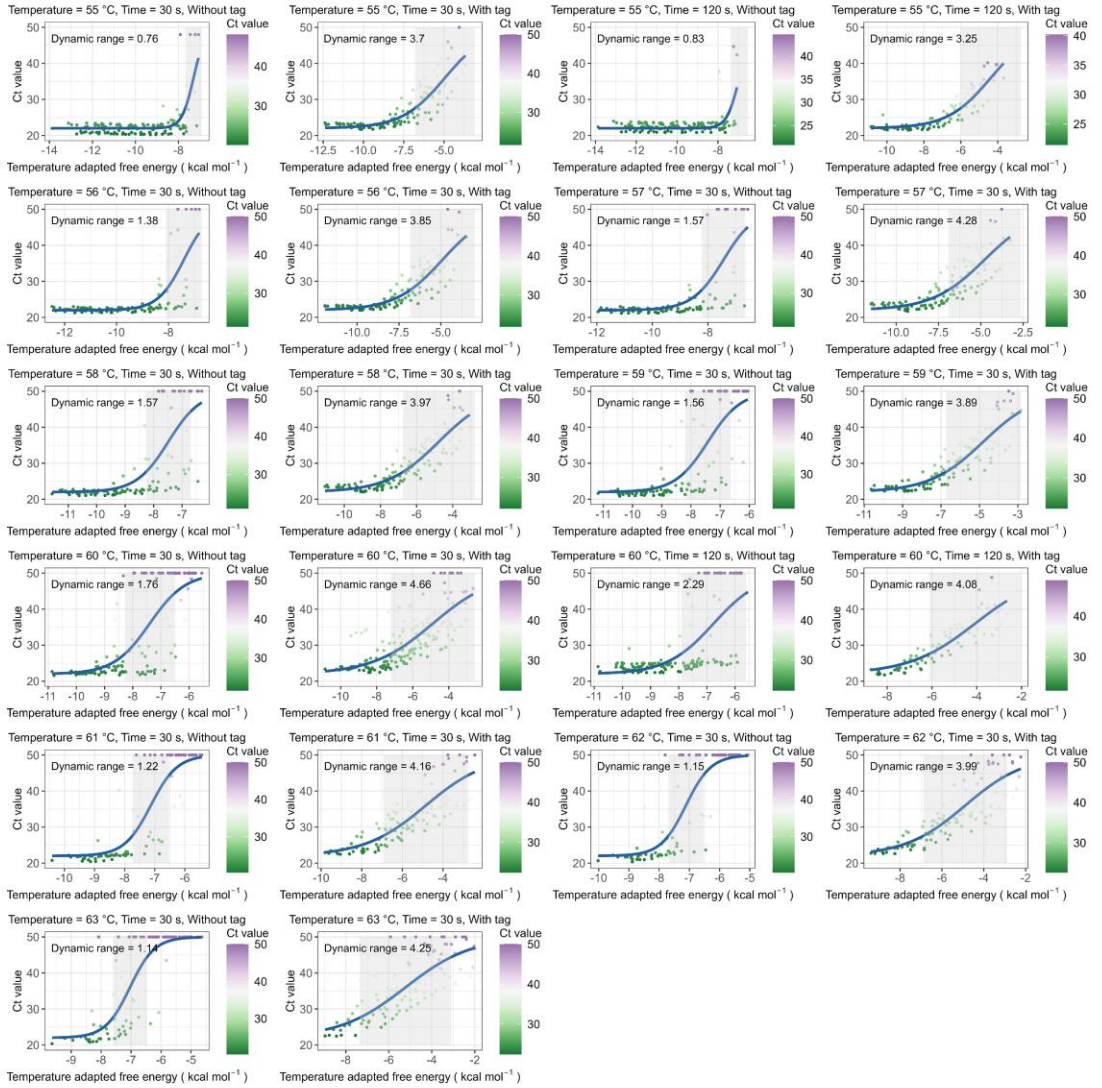

Fig. S10 | Scatter plots and logistic fits of Ct versus sequence free energy under different protocols.

Free energy values were calculated at the corresponding reaction temperature for each sequence. The relationship between Ct and temperature-adjusted free energy was fitted using a logistic function: $Ct = L + \frac{U-L}{1+\exp\left(\frac{x_{mid}-x}{scal}\right)}$, where L and U represent the lower and upper Ct bounds (fixed at 22 and 50, respectively), x_{mid} is the midpoint, and *scal* (fixed at 1) defines the slope. Nonlinear least-squares fitting was performed using the nlsLM algorithm, and the dynamic range (20–80% response interval) was calculated from the fitted curve.

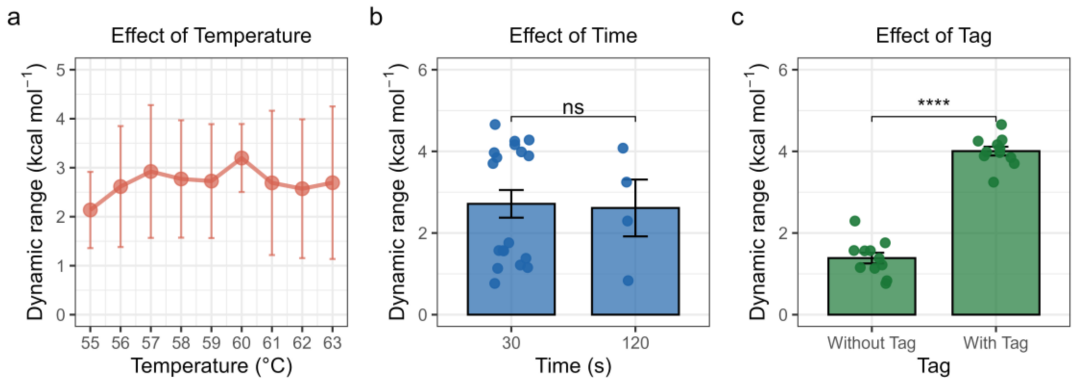

Fig. S11 | Effects of different factors on amplification dynamic range.

(a) Dynamic range across temperatures (55-63°C). Data represent mean ± s.e.m. (b) Dynamic range comparison between extension times (30 s vs. 120 s), showing negligible differences (two-sided *t*-test). (c) Impact of Tag sequences on dynamic range (two-sided *t*-test). Addition of Tag increased dynamic range from 1.39 to 4.01 kcal mol^{-1}. Dynamic range calculated as the ΔG span corresponding to 20-80% of the maximum response in the sigmoidal Ct vs. ΔG relationship.

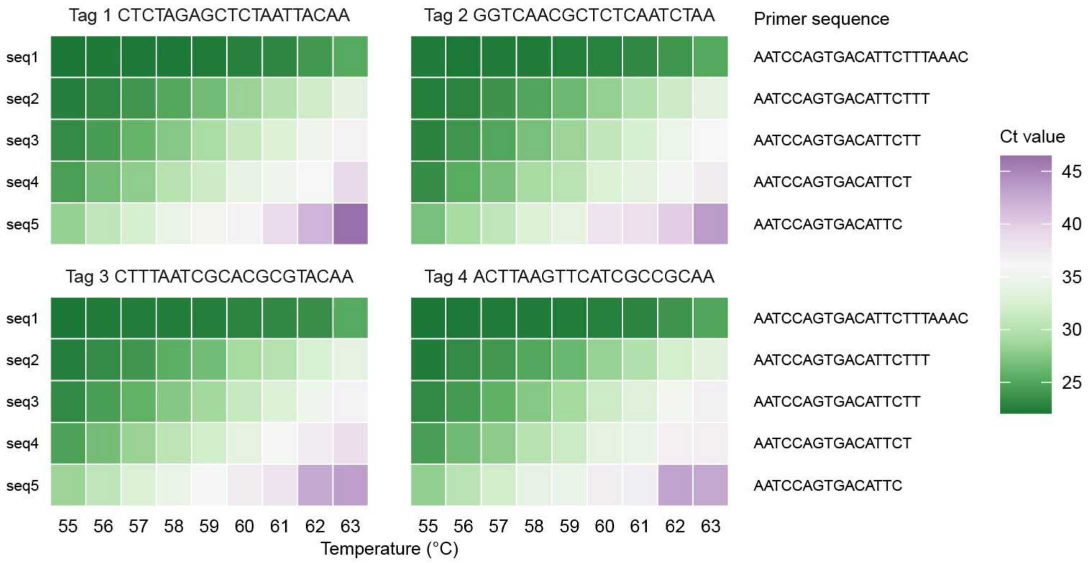

Fig. S12 | Generalizability of Tag-primer strategy across diverse Tag sequences.

Four distinct Tag sequences with different GC content were evaluated across multiple temperatures and primer lengths. Y-axis shows primer sequences ordered by increasing length (from bottom to top), with each primer progressively truncated from the 3' end. Ct values are represented by color intensity. Despite substantial sequence diversity among the four Tag sequences (shown in panel labels), all demonstrated consistent continuous tunability with gradual Ct modulation across temperatures and primer lengths. This sequence-independent performance confirms that PA relies on thermodynamic principles rather than specific sequence features, establishing broad applicability for primer design across diverse genomic contexts.

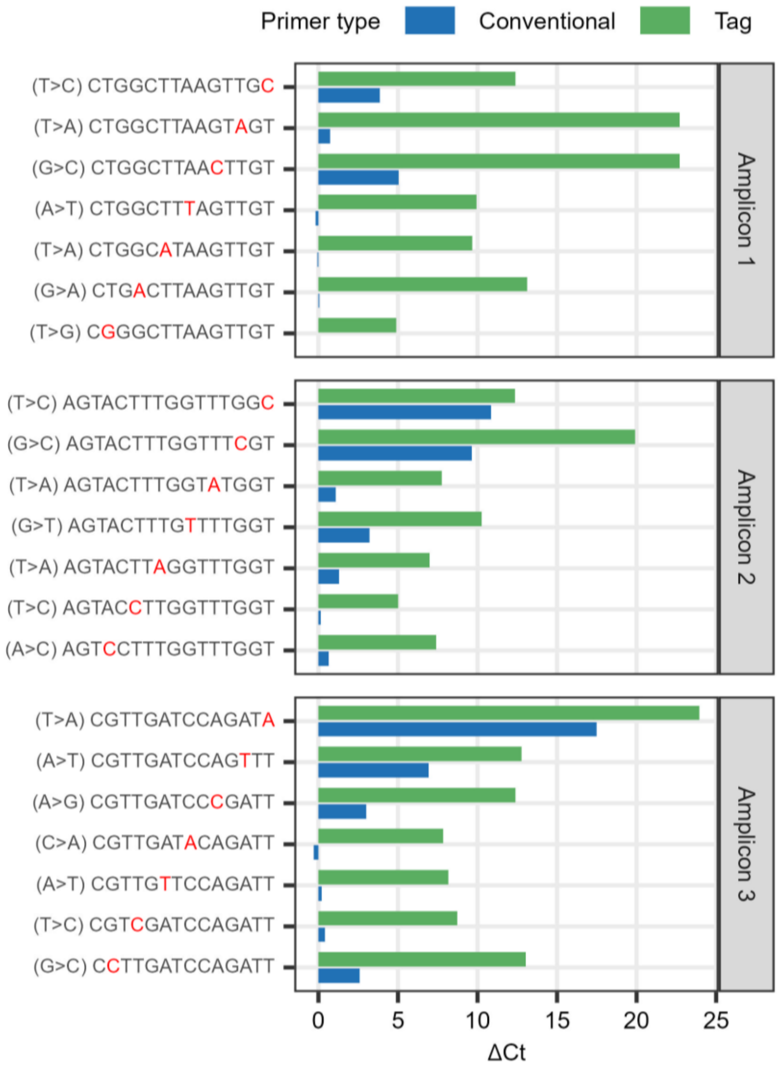

Fig. S13 | Single-nucleotide discrimination capability of conventional and Tag-primers.

Single-base mutations were introduced into primers to create mismatches with the template, enabling evaluation of primer specificity. ΔCt represents the difference in cycle threshold between mismatched and perfectly matched primers—larger ΔCt values indicate stronger discrimination against mismatched templates. Three amplicons and related primers were tested with various single-base substitutions at different positions. Y-axis labels show the mutation type and the primer sequence shared between conventional and Tag-primer designs, with mutated bases highlighted in red. Tag-primers consistently exhibited larger ΔCt values (mean: 12.0 cycles) compared to conventional primers (mean: 3.2 cycles), demonstrating superior specificity in distinguishing single-nucleotide variations. This enhanced discrimination stems from Tag-primers' truncated binding regions, which amplify the energetic penalty of mismatches across iterative PCR cycles.

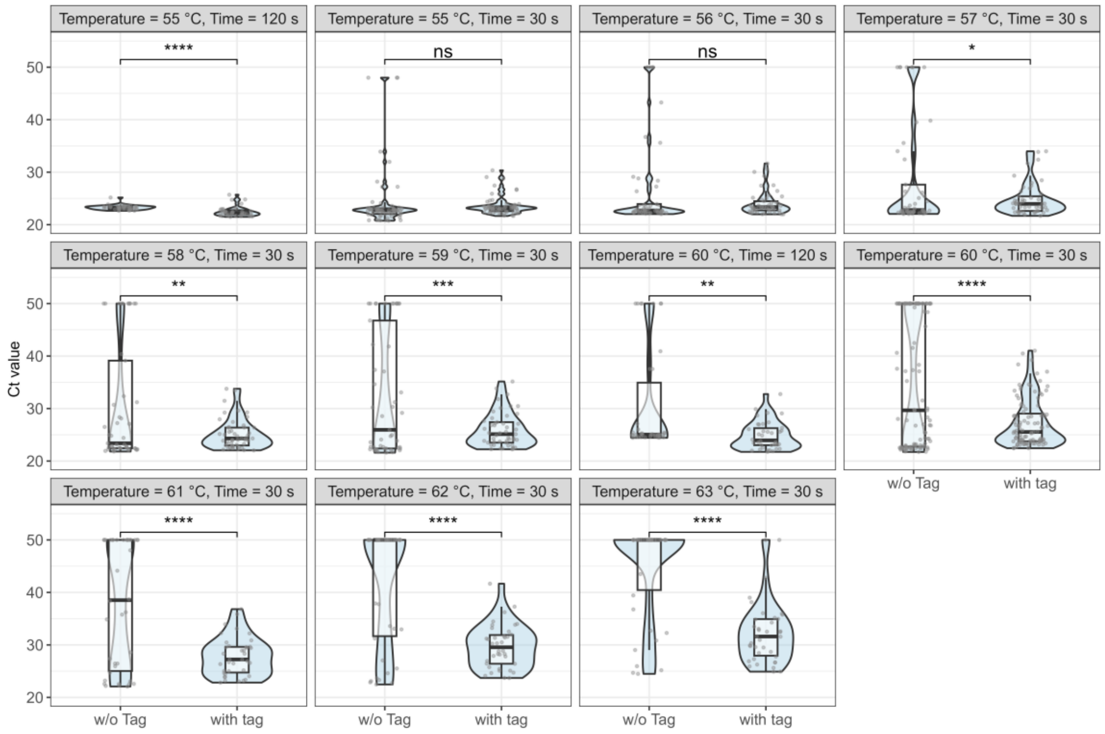

Fig. S14 | Comparison of Ct values between primers with and without tag sequences across combinations of reaction temperatures and extension times.

In most cases, adding a tag significantly reduced Ct values when the initial binding region was identical. For groups without significant differences, Ct values were already at their minimum without a tag. Statistical comparisons performed using two-sided t-test. Significance levels: ns, not significant; * $p < 0.05$; ** $p < 0.01$; *** $p < 0.001$; **** $p < 0.0001$.

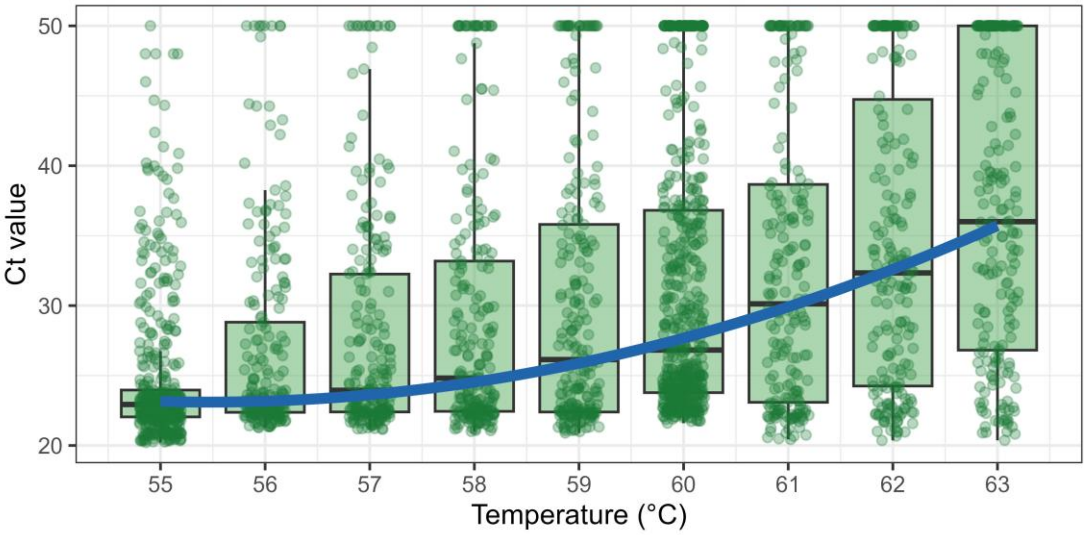

Fig. S15 | Distribution of Ct values under different reaction temperatures.

As the temperature increased, the median Ct value also rose, and the overall distribution became more dispersed.

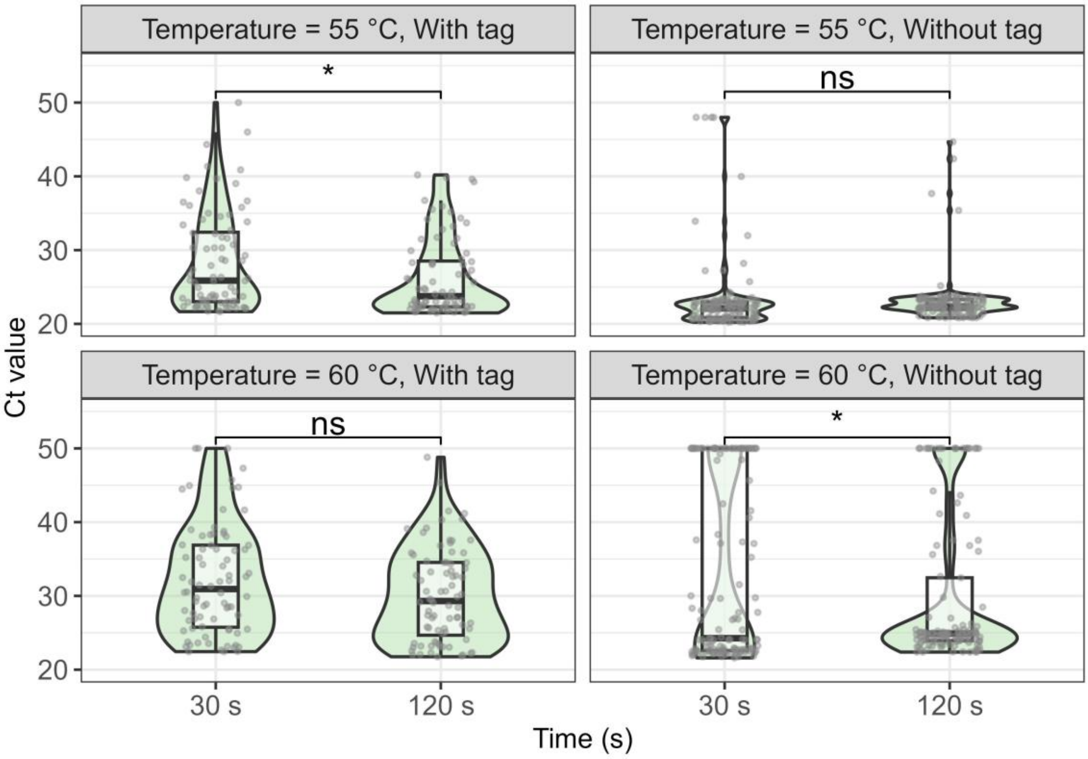

Fig. S16 | Effect of extension time on Ct values under different conditions.

Increasing the extension time from 30 s to 120 s had a relatively small impact on the distribution of Ct values; however, a slight overall decrease can still be observed. Statistical comparisons performed using two-sided t-test. Significance levels: ns, not significant; * $p < 0.05$; ** $p < 0.01$; *** $p < 0.001$; **** $p < 0.0001$.

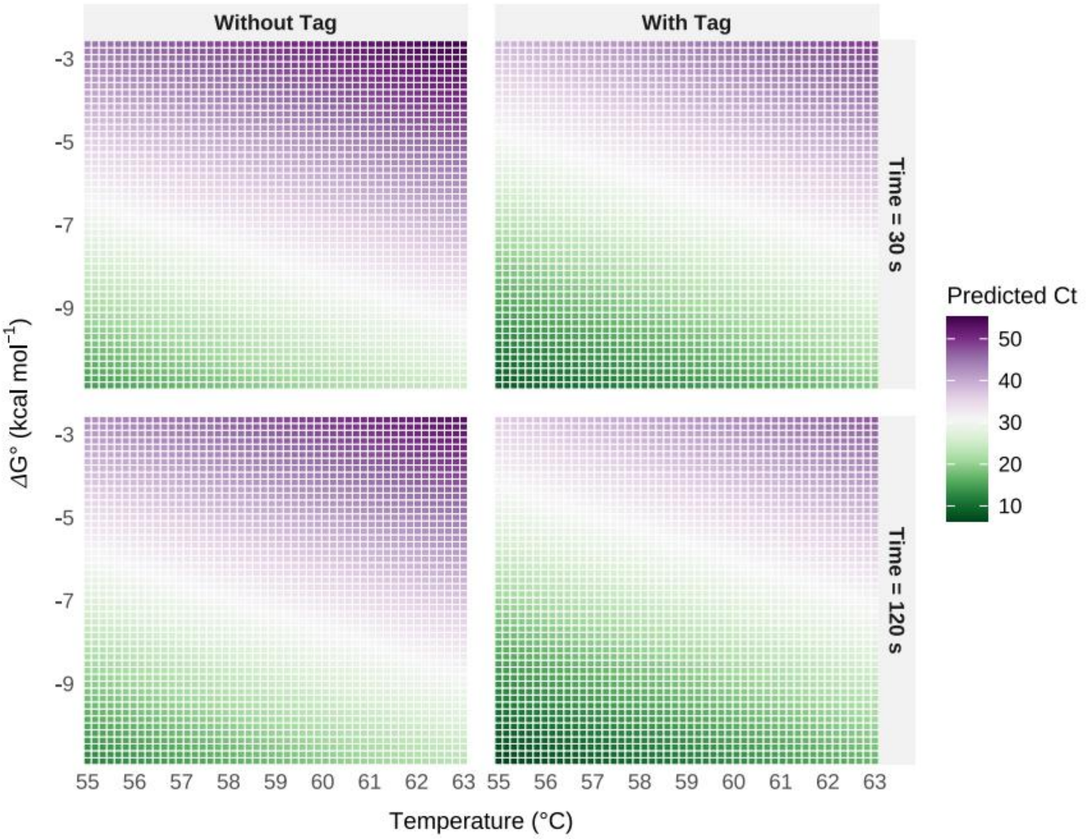

Fig. S17 | Predictions of amplification performance under different conditions using a linear model.

Overall, more negative free energy, lower temperatures, the presence of a tag, and longer extension times were associated with improved amplification efficiency and reduced Ct values.

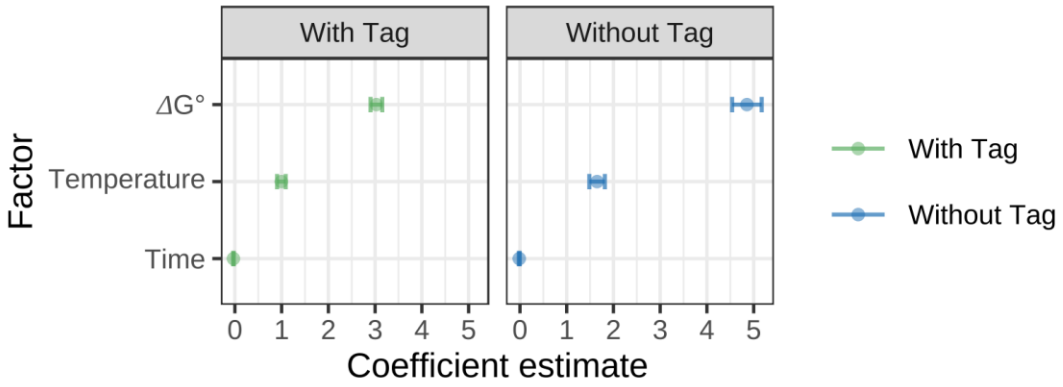

Fig. S18 | Parameter estimates of the linear model with and without tag sequences.

Tag incorporation markedly reduced the estimated coefficients for ΔG° and temperature, indicating decreased sensitivity to these factors and enabling a broader dynamic range.

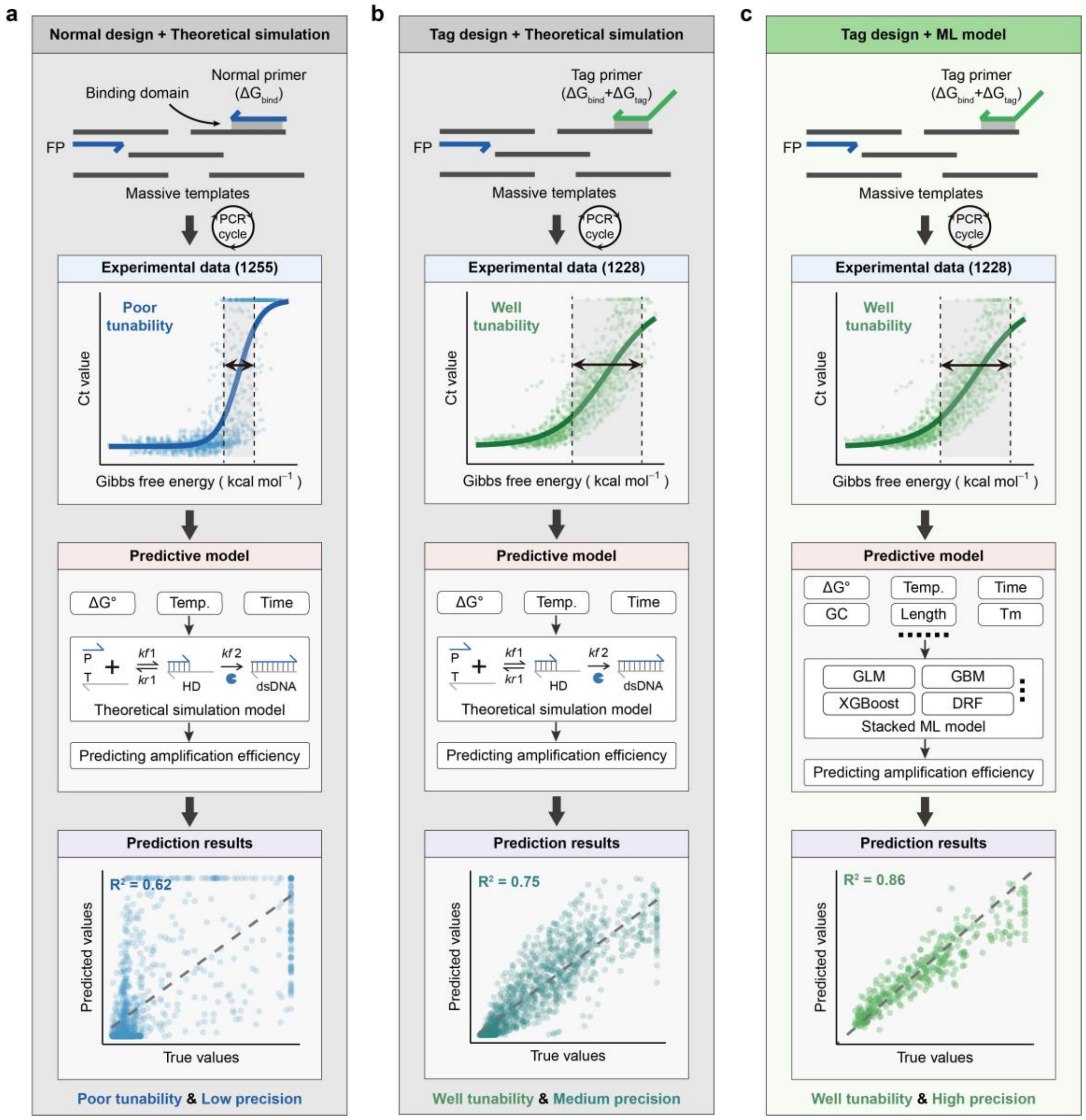

Fig. S19 | Precisely tunable nucleic acid amplification via Tag-primers and machine learning. (a) Performance of conventional primers using theoretical modeling. (b) Tag-primers with theoretical modeling: expanded dynamic range paired with limited predictive accuracy, resulting in moderate tuning precision. (c) Tag-primers integrated with machine learning: combined dynamic range enhancement and predictive precision, achieving optimal tunability. Each datapoint represents experimental measurements from distinct primer sequences under varied protocols.

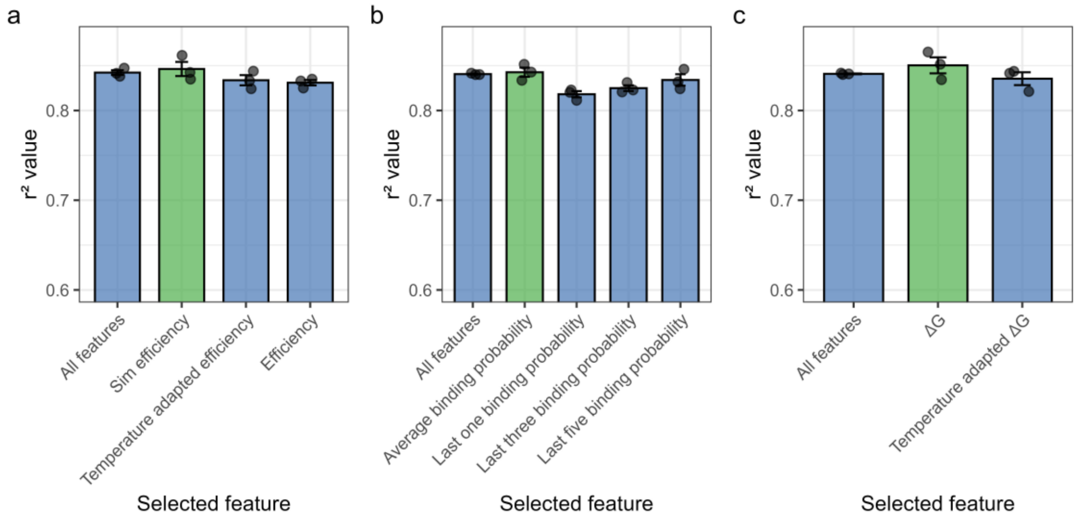

Fig. S20 | Feature ablation results for the stacked machine learning model.

(a) Efficiency-related features, (b) binding probability features, and (c) free-energy features were individually removed, with model performance compared to a baseline using all features. Error bars represent standard deviations from three independent experiments. The final selected features were Sim efficiency, Average binding probability, and ΔG.

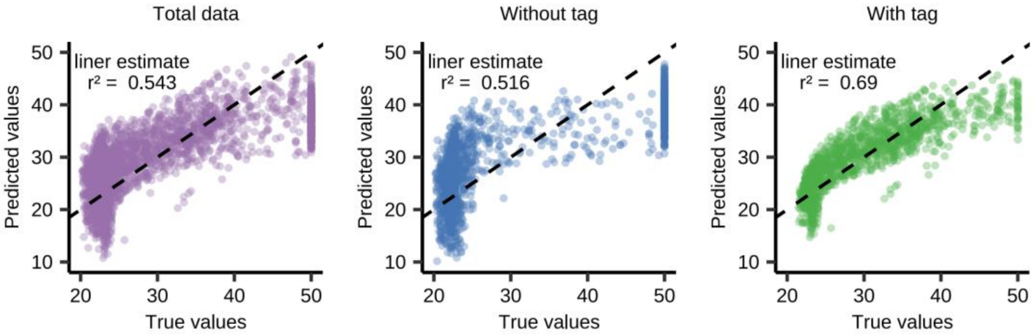

Fig. S21 | Linear model prediction performance across datasets.

Prediction accuracy improved progressively from the without-tag group ($r^2 = 0.516$) to the total dataset ($r^2 = 0.543$) and reached its highest level in the with-tag group ($r^2 = 0.690$), indicating that tag incorporation enhanced model predictability.

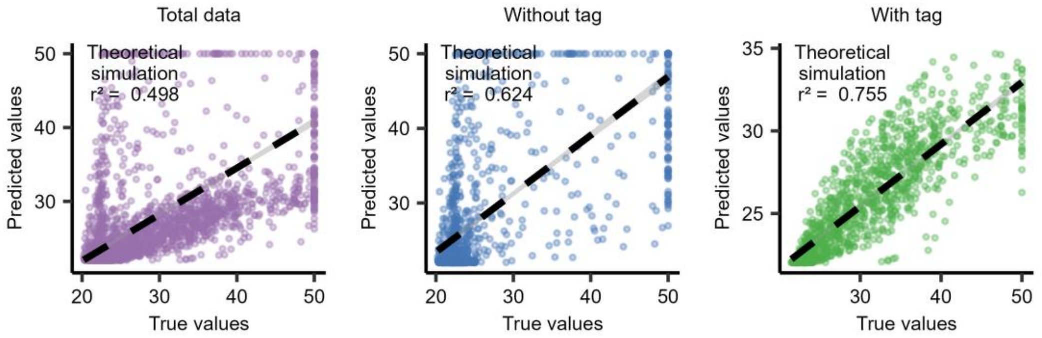

Fig. S22 | Theoretical model prediction performance across datasets.

Prediction accuracy increased from the total dataset ($r^2 = 0.498$) to the without-tag group ($r^2 = 0.624$) and reached its highest level in the with-tag group ($r^2 = 0.755$), suggesting that tag incorporation enhanced the predictive ability of the theoretical model.

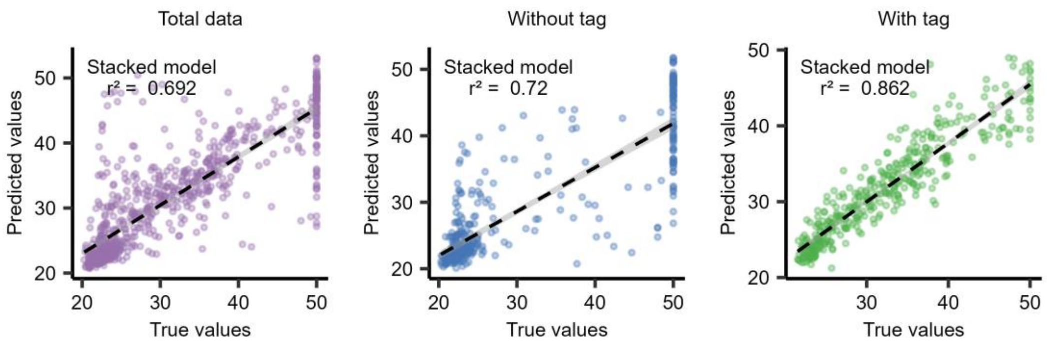

Fig. S23| Stacked model prediction performance across datasets.

Prediction accuracy increased from the total dataset ($r^2 = 0.692$) to the without-tag group ($r^2 = 0.72$) and reached its highest level in the with-tag group ($r^2 = 0.862$), suggesting that tag incorporation enhanced the predictive ability of the stacked model.

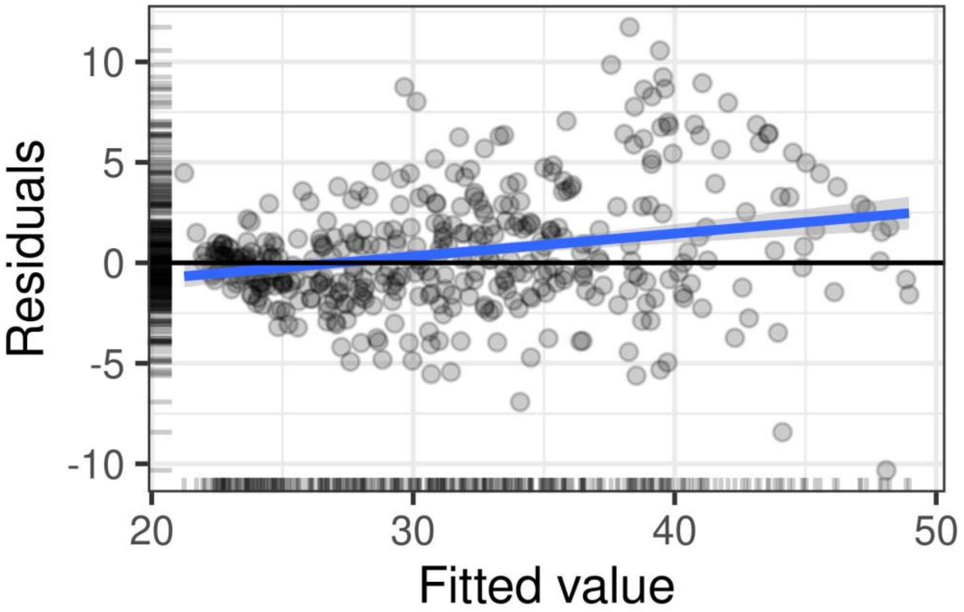

Fig. S24 | Residual distribution of predictions from the stacked model.

Eighty percent of residuals were within 4.5 cycles. Prediction accuracy was slightly lower for Ct values between 40 and 50, likely due to limited sample size in this range.

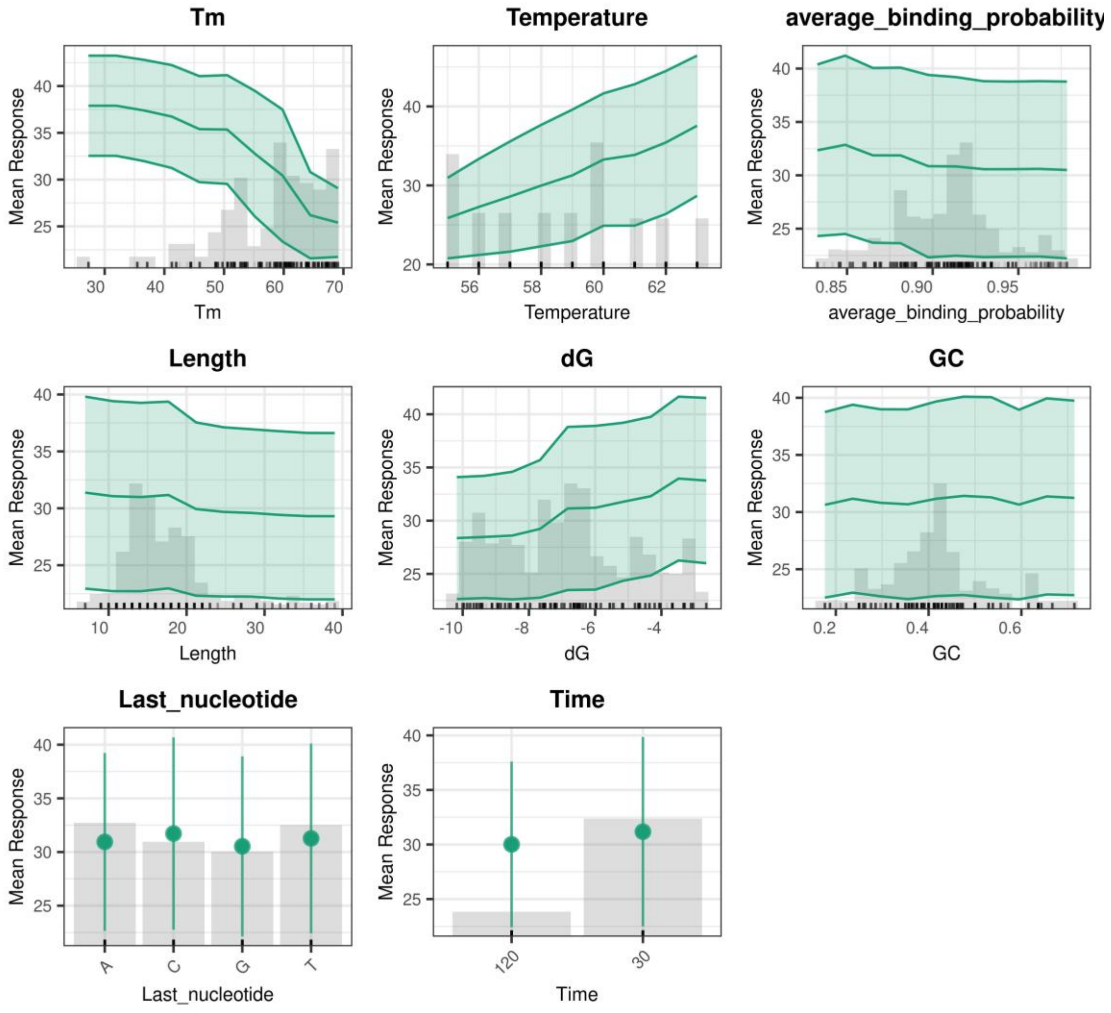

Fig. S25 | Individual conditional expectation (PDP) plots based on the stacked model.

Each subplot illustrates the marginal effect of one feature on the predicted Ct value while holding other features constant. Curves capture potential non-linear relationships between features and predictions, with shaded regions representing uncertainty intervals. Analysis was performed on the test set (30% of the data).

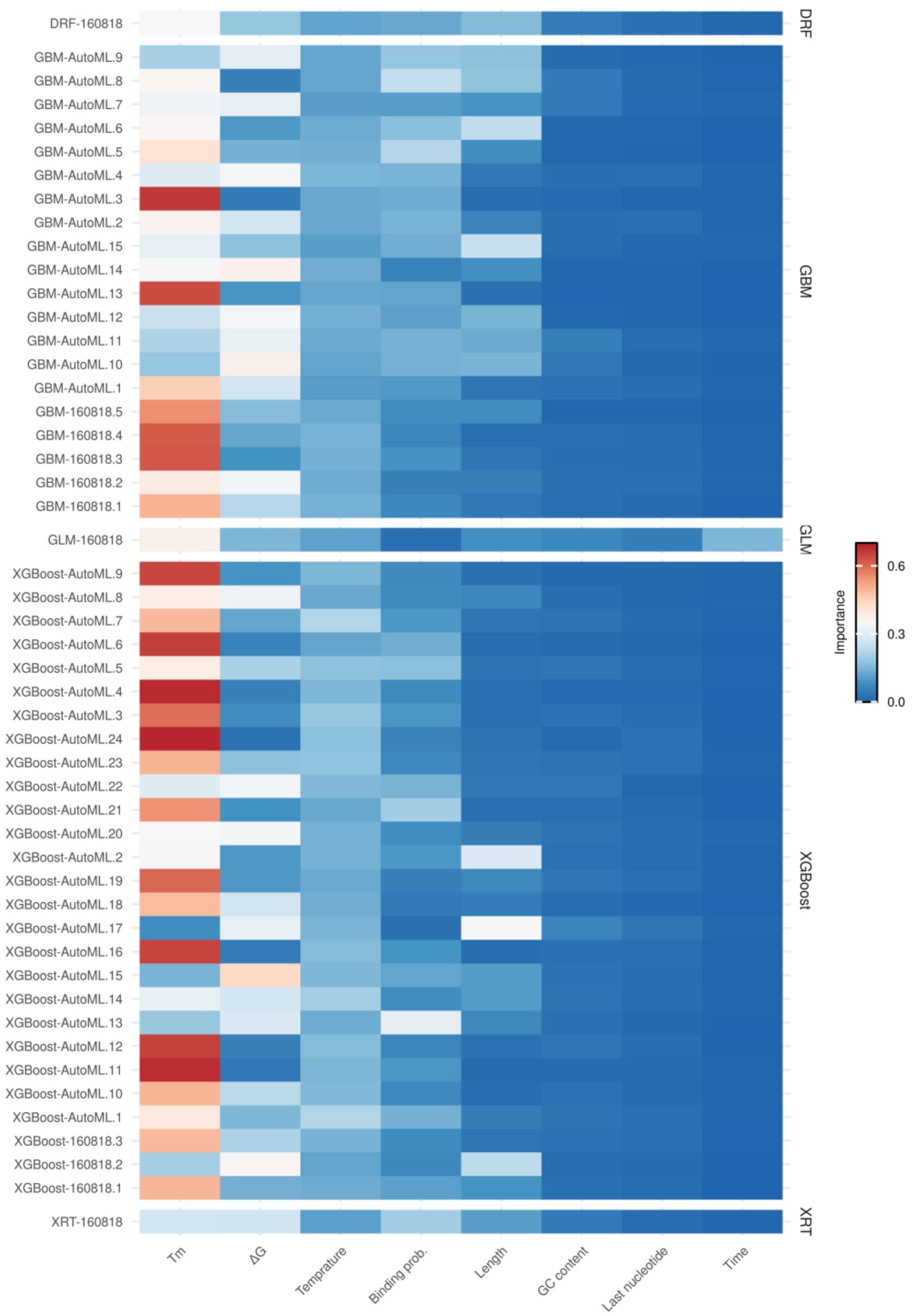

Fig. S26 | Feature importance heatmap across base models.

The heatmap illustrates the relative importance of key features for multiple base models generated during the AutoML process, grouped by model type. The horizontal axis lists the main features, including Tm, ΔG°, Temperature, Average binding probability, Length, GC content, Last nucleotide, and Time; the vertical axis represents individual models in their original ranking order. Color intensity denotes normalized feature importance, with red indicating high contribution and blue indicating low contribution.

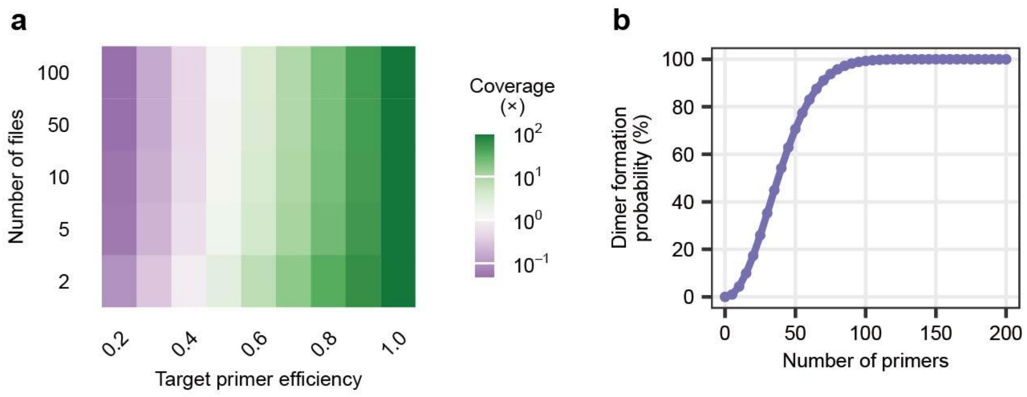

Fig. S27 | Scalability analysis of PA in multiplex scenarios.

(a) Coverage distribution of a target file with varying primer efficiency across different multiplex levels. Simulations assume 15 PCR cycles, 1000 initial copies, expected 100× depth per file, and other primers operating at 100% efficiency. (b) Primer dimer formation probability as a function of primer pool size. Calculations assume a 0.1% probability of dimer formation per primer pair.

Supplementary Table

Table S1 Primer sequences used for database construction

Primer name[a]	Primer sequences (5'→3')
M2-FP-Normal[b]	ACCAATGGGAGTCACTGCTG
M2-RP-Normal	TAAGTGGAAAGAACTGGGGTGTC
M7-FP-Normal	TACAAGGAAGCTCAGAGTAAAATTTACTG
M7-RP-Normal	AGTTTGGTACCTATTCCTCCAGGA
M8-FP-Normal	GGATTCCCTAAGCTCTTCAATATTGC
M8-RP-Normal	AAATGTAGTTCTATTATGGTCAGCACAC
M10-FP-Normal	ACTTCTGCCAACATTCAAATTCAGG
M10-RP-Normal	GTGCAAGCTGGAGGCACT
M12-FP-Normal	GGATGGGACTCCAATGCAAAACT
M12-RP-Normal	ACAGGAAAAGAAACTAAAATTGTACCCTT
MP6-FP-Normal	CACTTTATCAGACACAGTTATGTGCT
MP6-RP-Normal	GCAAAAATTACTATACCGACTTTAATAACGAAA
MP12-FP-Normal	CAAAGTTGATAAATTAAAGGACTAAGGCAC
MP12-RP-Normal	ACTTACTGAGCACATGGCCTG
MP21-FP-Normal	TGAAAGACGTCACAGCAAGGT
MP21-RP-Normal	AATCCAGTGACATTCTTTAAACTGTCTT
M22-FP-Normal	TGTAGGAGAGATTGGGCTAGAGAG
MP22-RP-Normal	GCTGAGCTGTCACATCACTTCA
M2-FP1	ACCAATGGGAGTCACTGC
M2-FP2	ACCAATGGGAGTCACTG
M2-FP3	ACCAATGGGAGTCACT
M2-FP4	ACCAATGGGAGTCAC
M2-FP5	ACCAATGGGAGTCA
M2-FP6	ACCAATGGGAGTC
M2-FP7	ACCAATGGGAGT
M7-FP1	TACAAGGAAGCTCAGAGTAAAATT
M7-FP2	TACAAGGAAGCTCAGAGTAAAAT
M7-FP3	TACAAGGAAGCTCAGAGTAAAA
M7-FP4	TACAAGGAAGCTCAGAGTAAA
M7-FP5	TACAAGGAAGCTCAGAGTAA
M7-FP6	TACAAGGAAGCTCAGAGTA
M7-FP7	TACAAGGAAGCTCAGAGT
M7-FP8	TACAAGGAAGCTCAGAG
M7-FP9	TACAAGGAAGCTCAGA
M7-FP10	TACAAGGAAGCTCAG
M7-FP11	TACAAGGAAGCTCA
M8-FP1	GGATTCCCTAAGCTCTTCAATAT
M8-FP2	GGATTCCCTAAGCTCTTCAATA
M8-FP3	GGATTCCCTAAGCTCTTCAAT
M8-FP4	GGATTCCCTAAGCTCTTCAA
M8-FP5	GGATTCCCTAAGCTCTTCA
M8-FP6	GGATTCCCTAAGCTCTTC
M8-FP7	GGATTCCCTAAGCTCTT

Primer name[a]	Primer sequences (5'→3')
M8-FP8	GGATTCCCTAAGCTCT
M8-FP9	GGATTCCCTAAGCTC
M8-FP10	GGATTCCCTAAGCT
M8-FP11	GGATTCCCTAAGC
M10-FP1	ACTTCTGCCAACATTCAAATTC
M10-FP2	ACTTCTGCCAACATTCAAATT
M10-FP3	ACTTCTGCCAACATTCAAAT
M10-FP4	ACTTCTGCCAACATTCAAA
M10-FP5	ACTTCTGCCAACATTCAA
M10-FP6	ACTTCTGCCAACATTCA
M10-FP7	ACTTCTGCCAACATTC
M10-FP8	ACTTCTGCCAACATT
M10-FP9	ACTTCTGCCAACAT
M10-FP10	ACTTCTGCCAACA
M10-FP11	ACTTCTGCCAAC
M12-FP1	GGATGGGACTCCAATGC
M12-FP2	GGATGGGACTCCAATG
M12-FP3	GGATGGGACTCCAAT
M12-FP4	GGATGGGACTCCAA
M12-FP5	GGATGGGACTCCA
M12-FP6	GGATGGGACTCC
mp6-FP1	CACTTTATCAGACACAGTTATGTG
mp6-FP2	CACTTTATCAGACACAGTTATGT
mp6-FP3	CACTTTATCAGACACAGTTATG
mp6-FP4	CACTTTATCAGACACAGTTAT
mp6-FP5	CACTTTATCAGACACAGTTA
mp6-FP6	CACTTTATCAGACACAGTT
mp6-FP7	CACTTTATCAGACACAGT
mp6-FP8	CACTTTATCAGACACAG
mp6-FP9	CACTTTATCAGACACA
mp12-FP1	CAAAGTTGATAAATTAAAGGACTAAGGC
mp12-FP2	CAAAGTTGATAAATTAAAGGACTAAGG
mp12-FP3	CAAAGTTGATAAATTAAAGGACTAAG
mp12-FP4	CAAAGTTGATAAATTAAAGGACTAA
mp12-FP5	CAAAGTTGATAAATTAAAGGACTA
mp12-FP6	CAAAGTTGATAAATTAAAGGACT
mp12-FP7	CAAAGTTGATAAATTAAAGGAC
mp12-FP8	CAAAGTTGATAAATTAAAGGA
mp12-FP9	CAAAGTTGATAAATTAAAGG
mp21-FP1	TGAAAGACGTCACAGCAAG
mp21-FP2	TGAAAGACGTCACAGCAA
mp21-FP3	TGAAAGACGTCACAGCA
mp21-FP4	TGAAAGACGTCACAGC
mp21-FP5	TGAAAGACGTCACAG
mp21-FP6	TGAAAGACGTCACA
mp21-FP7	TGAAAGACGTCAC
Mp22-FP1	TGTAGGAGAGATTGGGCTAG

Primer name[a]	Primer sequences (5'→3')
Mp22-FP2	TGTAGGAGAGATTGGGCTA
Mp22-FP3	TGTAGGAGAGATTGGGCT
Mp22-FP4	TGTAGGAGAGATTGGGC
Mp22-FP5	TGTAGGAGAGATTGGG
Mp22-FP6	TGTAGGAGAGATTGG
M2-RP1	TAAGTGGAAAGAACTGGGGT
M2-RP2	TAAGTGGAAAGAACTGGGG
M2-RP3	TAAGTGGAAAGAACTGGG
M2-RP4	TAAGTGGAAAGAACTGG
M2-RP5	TAAGTGGAAAGAACTG
M7-RP1	AGTTTGGTACCTATTCCTCCA
M7-RP2	AGTTTGGTACCTATTCCTCC
M7-RP3	AGTTTGGTACCTATTCCTC
M7-RP4	AGTTTGGTACCTATTCCT
M7-RP5	AGTTTGGTACCTATTCC
M7-RP6	AGTTTGGTACCTATTC
M8-RP1	AAATGTAGTTCTATTATGGTCAGCA
M8-RP2	AAATGTAGTTCTATTATGGTCAGC
M8-RP3	AAATGTAGTTCTATTATGGTCAG
M8-RP4	AAATGTAGTTCTATTATGGTCA
M8-RP5	AAATGTAGTTCTATTATGGTC
M8-RP6	AAATGTAGTTCTATTATGGT
M10-RP1	GTGCAAGCTGGAGGC
M10-RP2	GTGCAAGCTGGAGG
M10-RP3	GTGCAAGCTGGAG
M10-RP4	GTGCAAGCTGGA
M10-RP5	GTGCAAGCTGG
M12-RP1	ACAGGAAAAGAAACTAAAATTGTACC
M12-RP2	ACAGGAAAAGAAACTAAAATTGTAC
M12-RP3	ACAGGAAAAGAAACTAAAATTGTA
M12-RP4	ACAGGAAAAGAAACTAAAATTGT
M12-RP5	ACAGGAAAAGAAACTAAAATTG
M12-RP6	ACAGGAAAAGAAACTAAAATT
M12-RP7	ACAGGAAAAGAAACTAAAAT
M12-RP8	ACAGGAAAAGAAACTAAAA
M12-RP9	ACAGGAAAAGAAACTAAA
MP6-RP1	GCAAAAATTACTATACCGACTTTAATAAC
MP6-RP2	GCAAAAATTACTATACCGACTTTAATAA
MP6-RP3	GCAAAAATTACTATACCGACTTTAATA
MP6-RP4	GCAAAAATTACTATACCGACTTTAAT
MP6-RP5	GCAAAAATTACTATACCGACTTTAA
MP6-RP6	GCAAAAATTACTATACCGACTTTA
MP6-RP7	GCAAAAATTACTATACCGACTTT
MP6-RP8	GCAAAAATTACTATACCGACTT
MP6-RP9	GCAAAAATTACTATACCGAC
MP6-RP10	GCAAAAATTACTATACCGA
MP6-RP11	GCAAAAATTACTATACCG

Primer name[a]	Primer sequences (5’→3’)
MP12-RP2	ACTTACTGAGCACATGG
MP12-RP3	ACTTACTGAGCACATG
MP12-RP4	ACTTACTGAGCACAT
MP12-RP5	ACTTACTGAGCACA
MP21-RP2	AATCCAGTGACATTCTTTAAACT
MP21-RP3	AATCCAGTGACATTCTTTAAAC
MP21-RP4	AATCCAGTGACATTCTTT
MP21-RP5	AATCCAGTGACATTCTT
MP21-RP6	AATCCAGTGACATTCT
MP21-RP7	AATCCAGTGACATTC
MP22-RP2	GCTGAGCTGTCACATCA
MP22-RP3	GCTGAGCTGTCACATC
MP22-RP4	GCTGAGCTGTCACAT
MP22-RP5	GCTGAGCTGTCAC
MP22-RP6	GCTGAGCTGTCA
MP22-RP7	GCTGAGCTGTC
M2-FP3-tag	GCAAGCCCTCACGTAGCGAAACCAATGGGAGTCACT
M2-FP4-tag	GCAAGCCCTCACGTAGCGAAACCAATGGGAGTCAC
M2-FP5-tag	GCAAGCCCTCACGTAGCGAAACCAATGGGAGTCA
M2-FP6-tag	GCAAGCCCTCACGTAGCGAAACCAATGGGAGTC
M2-FP7-tag	GCAAGCCCTCACGTAGCGAAACCAATGGGAGT
M2-FP8-tag	GCAAGCCCTCACGTAGCGAAACCAATGGGAG
M2-FP9-tag	GCAAGCCCTCACGTAGCGAAACCAATGGGA
M2-FP10-tag	GCAAGCCCTCACGTAGCGAAACCAATGGG
M7-FP3-tag	GCAAGCCCTCACGTAGCGAATACAAGGAAGCTCAGAGTAAAA
M7-FP4-tag	GCAAGCCCTCACGTAGCGAATACAAGGAAGCTCAGAGTAAA
M7-FP5-tag	GCAAGCCCTCACGTAGCGAATACAAGGAAGCTCAGAGTAA
M7-FP6-tag	GCAAGCCCTCACGTAGCGAATACAAGGAAGCTCAGAGTA
M7-FP7-tag	GCAAGCCCTCACGTAGCGAATACAAGGAAGCTCAGAGT
M7-FP8-tag	GCAAGCCCTCACGTAGCGAATACAAGGAAGCTCAGAG
M7-FP9-tag	GCAAGCCCTCACGTAGCGAATACAAGGAAGCTCAGA
M7-FP10-tag	GCAAGCCCTCACGTAGCGAATACAAGGAAGCTCAG
M7-FP11-tag	GCAAGCCCTCACGTAGCGAATACAAGGAAGCTCA
M7-FP12-tag	GCAAGCCCTCACGTAGCGAATACAAGGAAGCTC
M7-FP13-tag	GCAAGCCCTCACGTAGCGAATACAAGGAAGCT
M7-FP14-tag	GCAAGCCCTCACGTAGCGAATACAAGGAAGC
M8-FP4-tag	GCAAGCCCTCACGTAGCGAAGGATTCCCTAAGCTCTTCAA
M8-FP5-tag	GCAAGCCCTCACGTAGCGAAGGATTCCCTAAGCTCTTCA
M8-FP6-tag	GCAAGCCCTCACGTAGCGAAGGATTCCCTAAGCTCTTC
M8-FP7-tag	GCAAGCCCTCACGTAGCGAAGGATTCCCTAAGCTCTT
M8-FP8-tag	GCAAGCCCTCACGTAGCGAAGGATTCCCTAAGCTCT
M8-FP9-tag	GCAAGCCCTCACGTAGCGAAGGATTCCCTAAGCTC
M8-FP10-tag	GCAAGCCCTCACGTAGCGAAGGATTCCCTAAGCT
M8-FP11-tag	GCAAGCCCTCACGTAGCGAAGGATTCCCTAAGC
M8-FP12-tag	GCAAGCCCTCACGTAGCGAAGGATTCCCTAAG
M8-FP13-tag	GCAAGCCCTCACGTAGCGAAGGATTCCCTAA
M10-FP1-tag	GCAAGCCCTCACGTAGCGAAACTTCTGCCAACATTCAAATTC

Primer name[a]	Primer sequences (5'→3')
M10-FP2-tag	GCAAGCCCTCACGTAGCGAAACTTCTGCCAACATTCAAATT
M10-FP3-tag	GCAAGCCCTCACGTAGCGAAACTTCTGCCAACATTCAAAT
M10-FP4-tag	GCAAGCCCTCACGTAGCGAAACTTCTGCCAACATTCAAA
M10-FP5-tag	GCAAGCCCTCACGTAGCGAAACTTCTGCCAACATTCAA
M10-FP6-tag	GCAAGCCCTCACGTAGCGAAACTTCTGCCAACATTCA
M10-FP7-tag	GCAAGCCCTCACGTAGCGAAACTTCTGCCAACATTC
M10-FP8-tag	GCAAGCCCTCACGTAGCGAAACTTCTGCCAACATT
M10-FP9-tag	GCAAGCCCTCACGTAGCGAAACTTCTGCCAACAT
M10-FP10-tag	GCAAGCCCTCACGTAGCGAAACTTCTGCCAACA
M10-FP11-tag	GCAAGCCCTCACGTAGCGAAACTTCTGCCAAC
M10-FP12-tag	GCAAGCCCTCACGTAGCGAAACTTCTGCCAA
M10-FP13-tag	GCAAGCCCTCACGTAGCGAAACTTCTGCCA
M12-FP1-tag	GCAAGCCCTCACGTAGCGAAGGATGGGACTCCAATGC
M12-FP2-tag	GCAAGCCCTCACGTAGCGAAGGATGGGACTCCAATG
M12-FP3-tag	GCAAGCCCTCACGTAGCGAAGGATGGGACTCCAAT
M12-FP4-tag	GCAAGCCCTCACGTAGCGAAGGATGGGACTCCAA
M12-FP5-tag	GCAAGCCCTCACGTAGCGAAGGATGGGACTCCA
M12-FP6-tag	GCAAGCCCTCACGTAGCGAAGGATGGGACTCC
M12-FP7-tag	GCAAGCCCTCACGTAGCGAAGGATGGGACTC
M12-FP8-tag	GCAAGCCCTCACGTAGCGAAGGATGGGACT
M12-FP9-tag	GCAAGCCCTCACGTAGCGAAGGATGGGAC
mp6-FP1-tag	GCAAGCCCTCACGTAGCGAACACTTTATCAGACACAGTTATGTG
mp6-FP2-tag	GCAAGCCCTCACGTAGCGAACACTTTATCAGACACAGTTATGT
mp6-FP3-tag	GCAAGCCCTCACGTAGCGAACACTTTATCAGACACAGTTATG
mp6-FP4-tag	GCAAGCCCTCACGTAGCGAACACTTTATCAGACACAGTTAT
mp6-FP5-tag	GCAAGCCCTCACGTAGCGAACACTTTATCAGACACAGTTA
mp6-FP6-tag	GCAAGCCCTCACGTAGCGAACACTTTATCAGACACAGTT
mp6-FP7-tag	GCAAGCCCTCACGTAGCGAACACTTTATCAGACACAGT
mp6-FP8-tag	GCAAGCCCTCACGTAGCGAACACTTTATCAGACACAG
mp6-FP9-tag	GCAAGCCCTCACGTAGCGAACACTTTATCAGACACA
mp6-FP10-tag	GCAAGCCCTCACGTAGCGAACACTTTATCAGACAC
mp6-FP11-tag	GCAAGCCCTCACGTAGCGAACACTTTATCAGACA
mp6-FP12-tag	GCAAGCCCTCACGTAGCGAACACTTTATCAGAC
mp12-FP1-tag	GCAAGCCCTCACGTAGCGAACAAAGTTGATAAATTAAAGGACTAAGGC
mp12-FP2-tag	GCAAGCCCTCACGTAGCGAACAAAGTTGATAAATTAAAGGACTAAGG
mp12-FP3-tag	GCAAGCCCTCACGTAGCGAACAAAGTTGATAAATTAAAGGACTAAG
mp12-FP4-tag	GCAAGCCCTCACGTAGCGAACAAAGTTGATAAATTAAAGGACTAA
mp12-FP5-tag	GCAAGCCCTCACGTAGCGAACAAAGTTGATAAATTAAAGGACTA
mp12-FP6-tag	GCAAGCCCTCACGTAGCGAACAAAGTTGATAAATTAAAGGACT
mp12-FP7-tag	GCAAGCCCTCACGTAGCGAACAAAGTTGATAAATTAAAGGAC
mp12-FP8-tag	GCAAGCCCTCACGTAGCGAACAAAGTTGATAAATTAAAGGA
mp12-FP9-tag	GCAAGCCCTCACGTAGCGAACAAAGTTGATAAATTAAAGG
mp12-FP10-tag	GCAAGCCCTCACGTAGCGAACAAAGTTGATAAATTAAAG
mp12-FP11-tag	GCAAGCCCTCACGTAGCGAACAAAGTTGATAAATTAAA
mp21-FP1-tag	GCAAGCCCTCACGTAGCGAATGAAAGACGTCACAGCAAG
mp21-FP2-tag	GCAAGCCCTCACGTAGCGAATGAAAGACGTCACAGCAA
mp21-FP3-tag	GCAAGCCCTCACGTAGCGAATGAAAGACGTCACAGCA

Primer name[a]	Primer sequences (5'→3')
mp21-FP4-tag	GCAAGCCCTCACGTAGCGAATGAAAGACGTCACAGC
mp21-FP5-tag	GCAAGCCCTCACGTAGCGAATGAAAGACGTCACAG
mp21-FP6-tag	GCAAGCCCTCACGTAGCGAATGAAAGACGTCACA
mp21-FP7-tag	GCAAGCCCTCACGTAGCGAATGAAAGACGTCAC
mp21-FP8-tag	GCAAGCCCTCACGTAGCGAATGAAAGACGTCA
mp21-FP9-tag	GCAAGCCCTCACGTAGCGAATGAAAGACGTC
Mp22-FP1-tag	GCAAGCCCTCACGTAGCGAATGTAGGAGAGATTGGGCTAG
Mp22-FP2-tag	GCAAGCCCTCACGTAGCGAATGTAGGAGAGATTGGGCTA
Mp22-FP3-tag	GCAAGCCCTCACGTAGCGAATGTAGGAGAGATTGGGCT
Mp22-FP4-tag	GCAAGCCCTCACGTAGCGAATGTAGGAGAGATTGGGC
Mp22-FP5-tag	GCAAGCCCTCACGTAGCGAATGTAGGAGAGATTGGG
Mp22-FP6-tag	GCAAGCCCTCACGTAGCGAATGTAGGAGAGATTGG
Mp22-FP7-tag	GCAAGCCCTCACGTAGCGAATGTAGGAGAGATTG
Mp22-FP8-tag	GCAAGCCCTCACGTAGCGAATGTAGGAGAGATT
Mp22-FP9-tag	GCAAGCCCTCACGTAGCGAATGTAGGAGAGAT
M2-RP3-tag	GCAAGCCCTCACGTAGCGAATAAGTGGAAAGAACTGGG
M2-RP4-tag	GCAAGCCCTCACGTAGCGAATAAGTGGAAAGAACTGG
M2-RP5-tag	GCAAGCCCTCACGTAGCGAATAAGTGGAAAGAACTG
M2-RP6-tag	GCAAGCCCTCACGTAGCGAATAAGTGGAAAGAACT
M2-RP7-tag	GCAAGCCCTCACGTAGCGAATAAGTGGAAAGAAC
M2-RP8-tag	GCAAGCCCTCACGTAGCGAATAAGTGGAAAGAA
M2-RP9-tag	GCAAGCCCTCACGTAGCGAATAAGTGGAAAGA
M2-RP10-tag	GCAAGCCCTCACGTAGCGAATAAGTGGAAAG
M7-RP3-tag	GCAAGCCCTCACGTAGCGAAAGTTTGGTACCTATTCCTC
M7-RP4-tag	GCAAGCCCTCACGTAGCGAAAGTTTGGTACCTATTCCT
M7-RP5-tag	GCAAGCCCTCACGTAGCGAAAGTTTGGTACCTATTCC
M7-RP6-tag	GCAAGCCCTCACGTAGCGAAAGTTTGGTACCTATTC
M7-RP7-tag	GCAAGCCCTCACGTAGCGAAAGTTTGGTACCTATT
M7-RP8-tag	GCAAGCCCTCACGTAGCGAAAGTTTGGTACCTAT
M7-RP9-tag	GCAAGCCCTCACGTAGCGAAAGTTTGGTACCTA
M7-RP10-tag	GCAAGCCCTCACGTAGCGAAAGTTTGGTACCT
M7-RP11-tag	GCAAGCCCTCACGTAGCGAAAGTTTGGTACC
M7-RP12-tag	GCAAGCCCTCACGTAGCGAAAGTTTGGTAC
M8-RP3-tag	GCAAGCCCTCACGTAGCGAAAAATGTAGTTCTATTATGGTCAG
M8-RP4-tag	GCAAGCCCTCACGTAGCGAAAAATGTAGTTCTATTATGGTCA
M8-RP5-tag	GCAAGCCCTCACGTAGCGAAAAATGTAGTTCTATTATGGTC
M8-RP6-tag	GCAAGCCCTCACGTAGCGAAAAATGTAGTTCTATTATGGT
M8-RP7-tag	GCAAGCCCTCACGTAGCGAAAAATGTAGTTCTATTATGG
M8-RP8-tag	GCAAGCCCTCACGTAGCGAAAAATGTAGTTCTATTATG
M8-RP9-tag	GCAAGCCCTCACGTAGCGAAAAATGTAGTTCTATTAT
M8-RP10-tag	GCAAGCCCTCACGTAGCGAAAAATGTAGTTCTATTA
M10-RP2-tag	GCAAGCCCTCACGTAGCGAAGTGCAAGCTGGAGG
M10-RP3-tag	GCAAGCCCTCACGTAGCGAAGTGCAAGCTGGAG
M10-RP4-tag	GCAAGCCCTCACGTAGCGAAGTGCAAGCTGGA
M10-RP5-tag	GCAAGCCCTCACGTAGCGAAGTGCAAGCTGG
M10-RP6-tag	GCAAGCCCTCACGTAGCGAAGTGCAAGCTG
M10-RP7-tag	GCAAGCCCTCACGTAGCGAAGTGCAAGCT

Primer name[a]	Primer sequences (5'→3')
M10-RP8-tag	GCAAGCCCTCACGTAGCGAAGTGCAAGC
M12-RP4-tag	GCAAGCCCTCACGTAGCGAAACAGGAAAAGAAACTAAAATTGT
M12-RP5-tag	GCAAGCCCTCACGTAGCGAAACAGGAAAAGAAACTAAAATTG
M12-RP6-tag	GCAAGCCCTCACGTAGCGAAACAGGAAAAGAAACTAAAATT
M12-RP7-tag	GCAAGCCCTCACGTAGCGAAACAGGAAAAGAAACTAAAAT
M12-RP8-tag	GCAAGCCCTCACGTAGCGAAACAGGAAAAGAAACTAAAA
M12-RP9-tag	GCAAGCCCTCACGTAGCGAAACAGGAAAAGAAACTAAA
M12-RP10-tag	GCAAGCCCTCACGTAGCGAAACAGGAAAAGAAACTAA
M12-RP11-tag	GCAAGCCCTCACGTAGCGAAACAGGAAAAGAAACTA
M12-RP12-tag	GCAAGCCCTCACGTAGCGAAACAGGAAAAGAAACT
M12-RP13-tag	GCAAGCCCTCACGTAGCGAAACAGGAAAAGAAAC
M12-RP14-tag	GCAAGCCCTCACGTAGCGAAACAGGAAAAGAAA
M12-RP15-tag	GCAAGCCCTCACGTAGCGAAACAGGAAAAGAA
M12-RP16-tag	GCAAGCCCTCACGTAGCGAAACAGGAAAAGA
M12-RP17-tag	GCAAGCCCTCACGTAGCGAAACAGGAAAAG
MP6-RP8-tag	GCAAGCCCTCACGTAGCGAAGCAAAAATTACTATACCGACTT
MP6-RP9-tag	GCAAGCCCTCACGTAGCGAAGCAAAAATTACTATACCGAC
MP6-RP10-tag	GCAAGCCCTCACGTAGCGAAGCAAAAATTACTATACCGA
MP6-RP11-tag	GCAAGCCCTCACGTAGCGAAGCAAAAATTACTATACCG
MP6-RP12-tag	GCAAGCCCTCACGTAGCGAAGCAAAAATTACTATACC
MP6-RP13-tag	GCAAGCCCTCACGTAGCGAAGCAAAAATTACTATAC
MP6-RP14-tag	GCAAGCCCTCACGTAGCGAAGCAAAAATTACTATA
MP12-RP2-tag	GCAAGCCCTCACGTAGCGAAACTTACTGAGCACATGG
MP12-RP3-tag	GCAAGCCCTCACGTAGCGAAACTTACTGAGCACATG
MP12-RP4-tag	GCAAGCCCTCACGTAGCGAAACTTACTGAGCACAT
MP12-RP5-tag	GCAAGCCCTCACGTAGCGAAACTTACTGAGCACA
MP12-RP6-tag	GCAAGCCCTCACGTAGCGAAACTTACTGAGCAC
MP12-RP7-tag	GCAAGCCCTCACGTAGCGAAACTTACTGAGCA
MP12-RP8-tag	GCAAGCCCTCACGTAGCGAAACTTACTGAGC
MP12-RP9-tag	GCAAGCCCTCACGTAGCGAAACTTACTGAG
MP21-RP3-tag	GCAAGCCCTCACGTAGCGAAAATCCAGTGACATTCTTTAAAC
MP21-RP4-tag	GCAAGCCCTCACGTAGCGAAAATCCAGTGACATTCTTT
MP21-RP5-tag	GCAAGCCCTCACGTAGCGAAAATCCAGTGACATTCTT
MP21-RP6-tag	GCAAGCCCTCACGTAGCGAAAATCCAGTGACATTCT
MP21-RP7-tag	GCAAGCCCTCACGTAGCGAAAATCCAGTGACATTC
MP21-RP8-tag	GCAAGCCCTCACGTAGCGAAAATCCAGTGACATT
MP21-RP9-tag	GCAAGCCCTCACGTAGCGAAAATCCAGTGACAT
MP21-RP10-tag	GCAAGCCCTCACGTAGCGAAAATCCAGTGACA
MP21-RP11-tag	GCAAGCCCTCACGTAGCGAAAATCCAGTGAC
MP21-RP12-tag	GCAAGCCCTCACGTAGCGAAAATCCAGTGA
MP22-RP4-tag	GCAAGCCCTCACGTAGCGAAGCTGAGCTGTCACAT
MP22-RP5-tag	GCAAGCCCTCACGTAGCGAAGCTGAGCTGTCAC
MP22-RP6-tag	GCAAGCCCTCACGTAGCGAAGCTGAGCTGTCA
MP22-RP7-tag	GCAAGCCCTCACGTAGCGAAGCTGAGCTGTC
MP22-RP8-tag	GCAAGCCCTCACGTAGCGAAGCTGAGCTGT
MP22-RP9-tag	GCAAGCCCTCACGTAGCGAAGCTGAGCTG
MP22-RP10-tag	GCAAGCCCTCACGTAGCGAAGCTGAGCT

Primer name[a]	Primer sequences (5'→3')
MP22-RP11-tag	GCAAGCCCTCACGTAGCGAAGCTGAGC

[a] Nine target loci were selected on the plasmid for primer design. At each locus, a series of primers were constructed by introducing base additions or deletions at the 3' terminus.

[b] In each set of amplification experiments, only one of the primers—either FP or RP—was tuned, while the other was kept as the normal primer.

Table S2 Sequences used in PA strategy experimental validation

Name	Sequences (5'→3')
MP21-RP3-tag1	CTCTAGAGCTCTAATTACAAAATCCAGTGACATTCTTTAAAC
MP21-RP4-tag1	CTCTAGAGCTCTAATTACAAAATCCAGTGACATTCTTT
MP21-RP5-tag1	CTCTAGAGCTCTAATTACAAAATCCAGTGACATTCTT
MP21-RP6-tag1	CTCTAGAGCTCTAATTACAAAATCCAGTGACATTCT
MP21-RP7-tag1	CTCTAGAGCTCTAATTACAAAATCCAGTGACATTC
MP21-RP3-tag2	GGTCAACGCTCTCAATCTAAAATCCAGTGACATTCTTTAAAC
MP21-RP4-tag2	GGTCAACGCTCTCAATCTAAAATCCAGTGACATTCTTT
MP21-RP5-tag2	GGTCAACGCTCTCAATCTAAAATCCAGTGACATTCTT
MP21-RP6-tag2	GGTCAACGCTCTCAATCTAAAATCCAGTGACATTCT
MP21-RP7-tag2	GGTCAACGCTCTCAATCTAAAATCCAGTGACATTC
MP21-RP3-tag3	CTTTAATCGCACGCGTACAAAATCCAGTGACATTCTTTAAAC
MP21-RP4-tag3	CTTTAATCGCACGCGTACAAAATCCAGTGACATTCTTT
MP21-RP5-tag3	CTTTAATCGCACGCGTACAAAATCCAGTGACATTCTT
MP21-RP6-tag3	CTTTAATCGCACGCGTACAAAATCCAGTGACATTCT
MP21-RP7-tag3	CTTTAATCGCACGCGTACAAAATCCAGTGACATTC
MP21-RP3-tag4	ACTTAAGTTCATCGCCGCAAAATCCAGTGACATTCTTTAAAC
MP21-RP4-tag4	ACTTAAGTTCATCGCCGCAAAATCCAGTGACATTCTTT
MP21-RP5-tag4	ACTTAAGTTCATCGCCGCAAAATCCAGTGACATTCTT
MP21-RP6-tag4	ACTTAAGTTCATCGCCGCAAAATCCAGTGACATTCT
MP21-RP7-tag4	ACTTAAGTTCATCGCCGCAAAATCCAGTGACATTC
AMP1-FP	TGTCCAGGGAGGTAGAGCAG
AMP1-RP1	AGCAGGCTGGCTTAAGTTGT
AMP1-RP2	AGCAGGCTGGCTTAAGTTGC
AMP1-RP3	AGCAGGCTGGCTTAAGTAGT
AMP1-RP4	AGCAGGCTGGCTTAACTTGT
AMP1-RP5	AGCAGGCTGGCTTTAGTTGT
AMP1-RP6	AGCAGGCTGGCATAAGTTGT
AMP1-RP7	AGCAGGCTGACTTAAGTTGT
AMP1-RP8	AGCAGGCGGGCTTAAGTTGT
AMP1-TAG-RP1	GCAAGCCCTCACGTAGCGAACTGGCTTAAGTTGT
AMP1-TAG-RP2	GCAAGCCCTCACGTAGCGAACTGGCTTAAGTTGC
AMP1-TAG-RP3	GCAAGCCCTCACGTAGCGAACTGGCTTAAGTAGT
AMP1-TAG-RP4	GCAAGCCCTCACGTAGCGAACTGGCTTAACTTGT
AMP1-TAG-RP5	GCAAGCCCTCACGTAGCGAACTGGCTTTAGTTGT

AMP1-TAG-RP6	GCAAGCCCTCACGTAGCGAACTGGCATAAGTTGT
AMP1-TAG-RP7	GCAAGCCCTCACGTAGCGAACTGACTTAAGTTGT
AMP1-TAG-RP8	GCAAGCCCTCACGTAGCGAACGGGCTTAAGTTGT
AMP2-FP	AAGCCATATCCAGGTTGCCC
AMP2-RP1	AGGCCAGTACTTTGGTTTGGT
AMP2-RP2	AGGCCAGTACTTTGGTTTGGC
AMP2-RP3	AGGCCAGTACTTTGGTTTCGT
AMP2-RP4	AGGCCAGTACTTTGGTATGGT
AMP2-RP5	AGGCCAGTACTTTGTTTTGGT
AMP2-RP6	AGGCCAGTACTTAGGTTTGGT
AMP2-RP7	AGGCCAGTACCTTGGTTTGGT
AMP2-RP8	AGGCCAGTCCTTTGGTTTGGT
AMP2-TAG-RP1	GCAAGCCCTCACGTAGCGAAAGTACTTTGGTTTGGT
AMP2-TAG-RP2	GCAAGCCCTCACGTAGCGAAAGTACTTTGGTTTGGC
AMP2-TAG-RP3	GCAAGCCCTCACGTAGCGAAAGTACTTTGGTTTCGT
AMP2-TAG-RP4	GCAAGCCCTCACGTAGCGAAAGTACTTTGGTATGGT
AMP2-TAG-RP5	GCAAGCCCTCACGTAGCGAAAGTACTTTGTTTTGGT
AMP2-TAG-RP6	GCAAGCCCTCACGTAGCGAAAGTACTTAGGTTTGGT
AMP2-TAG-RP7	GCAAGCCCTCACGTAGCGAAAGTACCTTGGTTTGGT
AMP2-TAG-RP8	GCAAGCCCTCACGTAGCGAAAGTCCTTTGGTTTGGT
AMP3-FP	TCAGTGTGAGGATGTCTAGGGT
AMP3-RP1	AGTGCGACGTTGATCCAGATT
AMP3-RP2	AGTGCGACGTTGATCCAGATA
AMP3-RP3	AGTGCGACGTTGATCCAGTTT
AMP3-RP4	AGTGCGACGTTGATCCCGATT
AMP3-RP5	AGTGCGACGTTGATACAGATT
AMP3-RP6	AGTGCGACGTTGTTCCAGATT
AMP3-RP7	AGTGCGACGTCGATCCAGATT
AMP3-RP8	AGTGCGACCTTGATCCAGATT
AMP3-TAG-RP1	GCAAGCCCTCACGTAGCGAACGTTGATCCAGATT
AMP3-TAG-RP2	GCAAGCCCTCACGTAGCGAACGTTGATCCAGATA
AMP3-TAG-RP3	GCAAGCCCTCACGTAGCGAACGTTGATCCAGTTT
AMP3-TAG-RP4	GCAAGCCCTCACGTAGCGAACGTTGATCCCGATT
AMP3-TAG-RP5	GCAAGCCCTCACGTAGCGAACGTTGATACAGATT
AMP3-TAG-RP6	GCAAGCCCTCACGTAGCGAACGTTGTTCCAGATT
AMP3-TAG-RP7	GCAAGCCCTCACGTAGCGAACGTCGATCCAGATT
AMP3-TAG-RP8	GCAAGCCCTCACGTAGCGAACCTTGATCCAGATT

Table S3 Sequences used in DNA storage system

Name	Sequences (5'→3')
ML-FP-Normal	ACCAATGGGAGTCACTGCTG
ML-RP-Normal	CGTACACTGGATCAGCGTCG
ML-FP-Normal-AD	TCGTCGGCAGCGTCAGATGTGTATAAGAGACAGACCAATGGGAGTCACTGCTG
ML-RP-Normal-AD	GTCTCGTGGGCTCGGAGATGTGTATAAGAGACAGCGTACACTGGATCAGCGTCG
SJTU-FP-Normal	GCTCTTCCTCTCACATCTTTATTTAACC
SJTU-RP-Normal	CACTGCCAGCTTGTGCCT
SJTU-FP-Normal-AD	TCGTCGGCAGCGTCAGATGTGTATAAGAGACAGGCTCTTCCTCTCACATCTTTATTTAACC
SJTU-RP-Normal-AD	GTCTCGTGGGCTCGGAGATGTGTATAAGAGACAGCACTGCCAGCTTGTGCCT
FR-FP-Normal	GGATGGGACTCCAATGCAAAACT
FR-RP-Normal	GAAGCCAGATCTCAAAGTGTCCT
FR-FP-Normal-AD	TCGTCGGCAGCGTCAGATGTGTATAAGAGACAGGGATGGGACTCCAATGCAAAACT
FR-RP-Normal-AD	GTCTCGTGGGCTCGGAGATGTGTATAAGAGACAGGAAGCCAGATCTCAAAGTGTCCT
ML-FP-tag-Preview	GCAAGCCCTCACGTAGCGAAACCAATGGGAGT
SJTU-FP-tag-Preview	GCAAGCCCTCACGTAGCGAAGCTCTTCCTCTCACAT
FR-FP-tag-Preview	GCAAGCCCTCACGTAGCGAAGGATGGGACTCCAA
ML-FP-lib1	ACCAATGGGAGTCACTGCTG
ML-FP-lib2	GCAAGCCCTCACGTAGCGAAACCAATGGGAGTCACTG
ML-FP-lib3	GCAAGCCCTCACGTAGCGAAACCAATGGGAGTCACT
ML-FP-lib4	GCAAGCCCTCACGTAGCGAAACCAATGGGAGTCAC
ML-FP-lib5	GCAAGCCCTCACGTAGCGAAACCAATGGGAGTCA
ML-FP-lib6	GCAAGCCCTCACGTAGCGAAACCAATGGGAGTC
SJTU-FP-steganography	GCAAGCCCTCACGTAGCGAAGCTCTTCCTCT
Tag-FP-AD	TCGTCGGCAGCGTCAGATGTGTATAAGAGACAGGCAAGCCCTCACGTAGCGAA
